# Developing organ dysfunction diagnostic criteria for children with cancer and post-hematopoietic cell transplantation: protocol of systematic review

**DOI:** 10.3389/fonc.2025.1591263

**Published:** 2025-10-15

**Authors:** Michael Bashir, Sayeda Islam, Jordan Wrigley, Anita Arias, Roelie M. Wösten-van Asperen, Kimberly K. Kertis, Melissa M. Hudson, Courtney M. Rowan, Aimee C. Talleur, Joshua Wolf, Benjamin Oelkers, Melissa R. Hines, Asya Agulnik

**Affiliations:** ^1^ Department of Global Pediatric Medicine, St Jude Children’s Research Hospital, Memphis, TN, United States; ^2^ Washington University in St. Louis School of Medicine, St. Louis, MO, United States; ^3^ Biomedical Library, St. Jude Children’s Research Hospital, Memphis, TN, United States; ^4^ Department of Pediatric Intensive Care, University Medical Centre Utrecht, Wilhelmina Children’s Hospital, Utrecht, Netherlands; ^5^ Division of Cancer Survivorship, Department of Oncology, St Jude Children’s Research Hospital, Memphis, TN, United States; ^6^ Department of Pediatrics, Division of Critical Care, Riley Hospital for Children, Indiana University School of Medicine, Indianapolis, IN, United States; ^7^ Department of Bone Marrow Transplantation and Cellular Therapy, St. Jude Children’s Research Hospital, Memphis, TN, United States; ^8^ Department of Infectious Diseases, St Jude Children’s Research Hospital, Memphis, TN, United States; ^9^ Department of Pediatrics, University of Tennessee Health Science Center, Memphis, TN, United States; ^10^ Division of Critical Care, Department of Pediatric Medicine, St Jude Children’s Research Hospital, Memphis, TN, United States

**Keywords:** multiorgan dysfunction, pediatric oncology, hematopoietic (stem cell) transplant (HSCT), critical care, PICU (pediatric intensive care unit), systematic reviews, protocol paper

## Abstract

**Background:**

The Pediatric Organ Dysfunction Information Update Mandate (PODIUM) proposed consensus criteria to define organ dysfunction in critically ill children. However, utilization of the PODIUM criteria in pediatric oncology patients and those who have received hematopoietic cell transplantation or cellular therapy (post-HCT/CAR) may inaccurately classify organ dysfunction in these patients due to differences in organ dysfunction etiology, pathophysiology, and risk factors for adverse outcomes. To address this gap, we report a study protocol to systematically review the performance of the PODIUM criteria for pediatric cancer and/or those treated with HCT and determine if adjustments are needed.

**Objective:**

The objectives of this study will be to [1] identify evidence-based criteria for organ dysfunction predicting adverse outcomes among pediatric oncology and post-HCT patients, [2] use these findings to inform adapted consensus criteria (PODIUM-Onc) for organ dysfunction tailored to this high-risk population through a multidisciplinary modified Delphi process, and [3] describe knowledge gaps to guide future research.

**Data source and search strategy:**

We will perform a systematic literature review of studies published since January 1, 2004, using the following databases: MEDLINE (via PubMed), CINAHL (via EBSCO), EMBASE (via Elsevier), and Web of Science (via Clarivate). Search results will be filtered using a pediatric search hedge and further refined to children (0 to 21 years old) during or up to 1 year after treatment for cancer or HCT/CAR for malignancy. Publications without original data (e.g., comments, editorials, letters, notes, conference materials), studies with ≤ 10 patients, and those preceding January 1, 2004, will be excluded.

**Study selection:**

We will include original studies in any language published since January 1, 2004, that meet all eligibility criteria and for which a full text is available.

**Data extraction:**

Data extraction will include information related to study characteristics, hospital characteristics, underlying population characteristics, patient population characteristics, and outcomes.

**Data synthesis:**

We will extract and report data on the study, hospital, and patient characteristics, outcomes, and risk of bias.

**Conclusion:**

By systematically reviewing and analyzing organ-specific factors associated with patient outcomes and synthesizing these findings through a modified Delphi consensus process, we aim to create consensus criteria that will be clinically relevant for pediatric oncology patients and HCT patients. These criteria will provide a foundation to guide clinical care and to support future research in this vulnerable patient population.

## Introduction

Multiple organ dysfunction (MOD) remains the leading cause of death in pediatric intensive care units (PICUs) and is a major contributor to both short- and long-term morbidity (1, 2). Children with cancer or those who have undergone hematopoietic cell transplantation (HCT) and/or cellular therapy (CAR) are especially vulnerable, facing an elevated risk of organ dysfunction and significantly higher mortality rates in the PICU (3–6). Mortality among pediatric cancer patients admitted to the PICU is reported to range from 23% to 28%, approximately five-fold higher than the overall PICU mortality of 5% (7, 8). Patients who develop MOD experience even greater risk, with mortality reported between 50% and 66%, depending on the study and timing of organ dysfunction onset (9, 10) The nature of organ dysfunction in pediatric oncology and post-HCT patients is distinct from that of other critically ill children or immunocompromised young adults, driven by unique etiologies, biological mechanisms, and mortality risk factors.

Historically, research on organ dysfunction in critically ill children has been limited by a lack of standardized organ dysfunction definitions. To address this challenge, in 2022 the Pediatric Organ Dysfunction Information Update Mandate (PODIUM) (3) published a consensus-driven set of criteria to standardize organ dysfunction definitions across 11 systems (cardiovascular, gastrointestinal, neurologic, hepatic, coagulation, endothelial, hematologic, respiratory, endocrine, renal, and immune), as well as for multiorgan dysfunction. Developed by an international expert panel informed by systematic literature reviews, these criteria aim to identify children with single or multiple organ dysfunction for research and to serve as entry criteria or outcome measures in clinical trials.

These general PODIUM criteria, however, are likely insufficient to characterize organ dysfunction among children treated for oncologic disorders, including those that receive allogeneic HCT or cellular therapy (5). First, this patient population is underrepresented in the literature reviewed by PODIUM, with only 2.2% (26 of 1,185) of studies including data from these patients (11). Key studies specifically addressing organ dysfunction in children with cancer were also absent (5, 12–15). Second, currently available scoring and severity-of-illness systems developed for the general pediatric population have limited performance in children with cancer (16, 17), and initial findings suggest the PODIUM criteria may face similar limitations (5). Finally, unique pathobiological factors drive organ dysfunction in this patient population (18, 19), stemming from intensive treatment regimens, such as chemotherapy or immunotherapy, HCT-related complications, or the underlying malignancy itself (20–22). For example, sinusoidal obstruction syndrome (SOS) and immune effector cell-associated cytokine release syndrome (CRS) are established forms of endothelial and immune dysfunction with specific severity grading criteria; however, neither is included in PODIUM.

To address these challenges, we have assembled an international panel of experts in pediatric onco-critical care to identify areas for adjustment of the current PODIUM organ dysfunction criteria for use in pediatric cancer and post-HCT patients, identify evidence-based markers of organ dysfunction that predict adverse outcomes and develop consensus criteria (PODIUM-Onc) tailored to this high-risk population using a multidisciplinary modified Delphi process. These criteria will guide clinical care and inform future research. The planned methodology is described in this protocol paper.

## Materials and methods

### Systematic review objectives

The objectives of this study are to conduct 12 linked systematic literature reviews to identify specific evidence-based criteria for defining organ dysfunction that predict an increased risk of adverse outcomes among pediatric cancer patients. This study is not a meta-analysis, and does not aim to generate clinical management recommendations. Rather, it seeks to establish a foundational framework upon which future work can validate, refine, and integrate these criteria to better identify children with single or multiple organ dysfunction and to characterize distinct patterns or phenotypes of organ dysfunction associated with poor outcomes.

### Protocol and registration

This protocol follows the Preferred Reporting Items for Systematic Review and Meta-Analysis Protocols guidelines (PRISMA) and is registered in the international Prospective Register of Systematic Reviews (PROSPERO 2024 CRD42024608850).

### Study team

**Figure 1:**
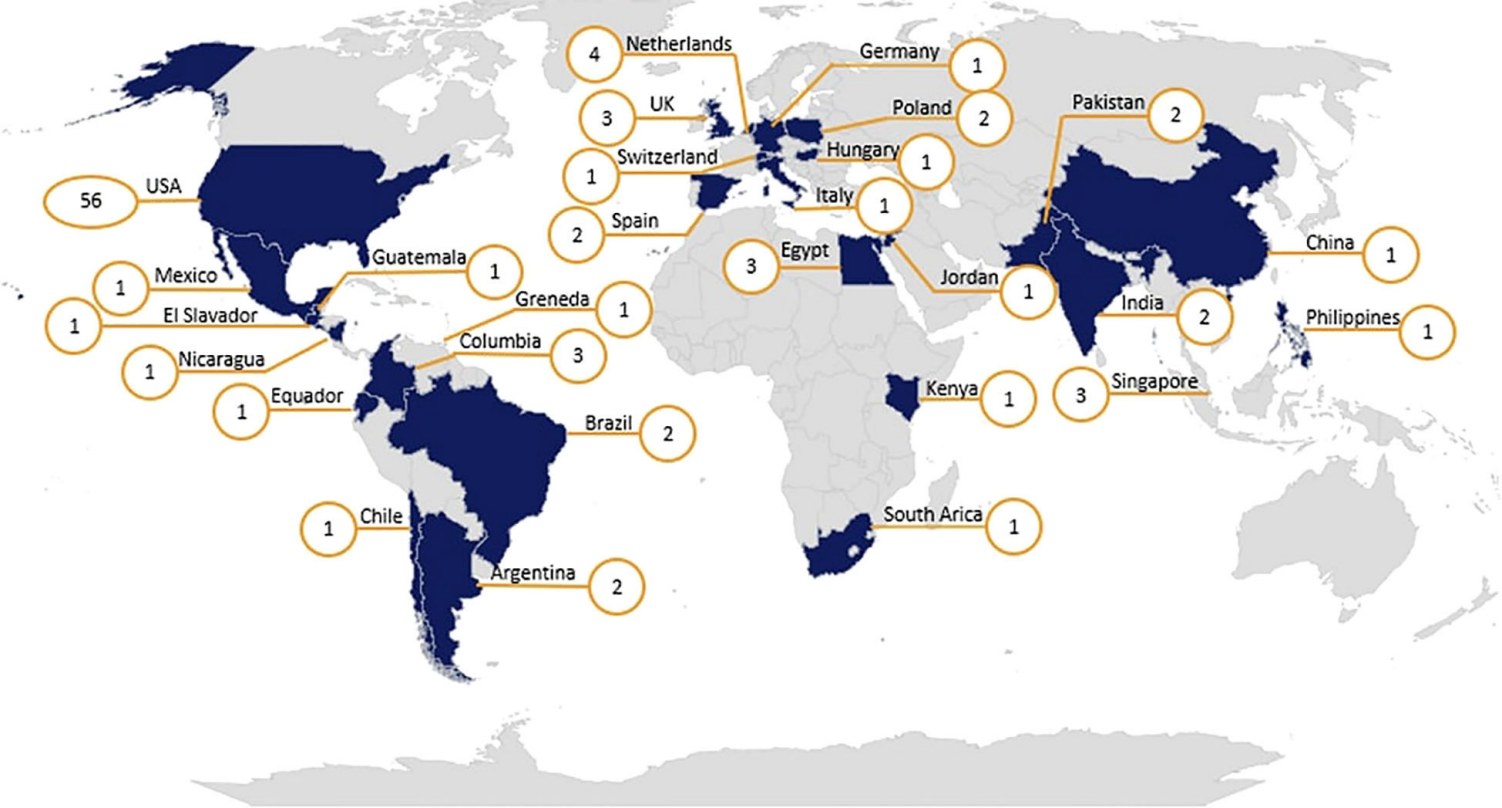
Global distribution of PODIUM-Onc collaborators. Numbers in orange circles represent the number of collaborators in each country.

This proposed work is led by a core team of diverse multidisciplinary experts in pediatric onco-critical care, hematology-oncology, HCT/CAR, and infectious disease, including an MPI team (AAg, MHi), six co-investigators (AAr, MHu, CR, AT, RWA, JWo), four program coordinators/trainees (MB, NBe, SI, BO), a research librarian (JWr), and a data management librarian (KK). This core team is supported by organ-system specific Working Groups comprised of 10–20 experts in the relevant fields (some investigators are included in multiple reviews as per their areas of expertise). The Working Groups were identified to be inclusive of disciplines (oncology, ICU, etc.), professions (nurses, physicians, respiratory therapists, etc.), income level of the country of practice, and to represent all World Health Organization (WHO) regions. Additionally, members of the team are affiliated with multiple professional organizations, including the Pediatric Acute Lung Injury and Sepsis Investigators (PALISI), the European Society of Paediatric and Neonatal Intensive Care (ESPNIC), the Pediatric Acute & Critical Care Medicine Asian Network (PACCMAN), the Sociedad Latinoamericana de Cuidados Intensivos Pediátricos (SLACIP), the Sociedad Latino Americana de Oncología Pediátrica (SLAOP), the Children’s Oncology Group(COG), the Pediatric Transplantation and Cellular Therapy Consortium (PTCTC), and the International Society of Paediatric Oncology (SIOP). This team also includes people with lived experience of cancer, including 2 childhood cancer survivors. These survivors are integral members of our study team and will actively participate in the data synthesis and modified Delphi consensus process alongside clinical experts, contributing as equal partners and co-authors on resulting manuscripts; their involvement in the systematic reviews will be guided by their individual interests and expertise. The modified Delphi panel will be used to reconcile evidence and achieve consensus on the adapted PODIUM-Onc criteria. **Table 1** summarizes the combined demographics of the current Core Team [14] and Working Group Members [90]. **Figure 1** illustrates the global distribution of PODIUM-Onc collaborators.

**Table 1:** Core team and working group demographics.

Participant Characteristics	n=104	%
Discipline *		
Pediatric ICU/Critical Care	57	47.5
Pediatric Oncology	17	14.2
General Medicine	3	5.8
Pediatric Oncology/HCT	6	5
Pediatric Endocrinology	6	5
Pediatric Hospitalist	6	5
Program Coordination	5	4.1
Pediatric Cardiology	2	1.7
Pediatric Nephrology	2	1.7
Pediatric Neurology	2	1.7
Patient Representative	2	1.7
Pediatric Pulmonology	1	0.8
Other	7	5.8
Profession		
Physician	89	85.6
Program Coordination	5	4.8
Research Librarian	2	1.9
Nurse Practitioner	2	1.9
Advanced Care Practitioner	1	1
Respiratory Therapist	1	1
Other	4	3.8
Total	104	100
Country Income Level		
High Income	77	77
Upper Middle Income	15	14.4
Lower Middle Income	12	11.5
Total	104	100
WHO Region		
AMRO	545	52
EURO	17	16.3
AMRO-LA	16	15.4
EMRO	8	7.7
WPRO	5	4.8
SEARO	2	1.9
AFRO	2	1.9
Total	104	100

* Choose all that apply options.

ICU, Intensive Care Unit; HCT, Hematopoietic Cell Transplantation; WHO, World Health Organization; AFRO, African Region; AMRO, The Region of the Americas; AMRO-LA, The Region of the Americas-Latin America; EURO, European Region; SEARO, South-East Asia Region; EMRO, Eastern Mediterranean Region; WPRO, Western Pacific Region.

### Population

The population of interest includes children, adolescents, and young adults aged 0–21 years diagnosed with cancer who experience organ dysfunction at any point before, during, or after hematopoietic cell transplantation (HCT), provided the indication for HCT is malignancy, presenting with either single or multiple episodes of acute organ failure within 1 year of completing cancer treatment. “Cancer” is defined as a malignant disease. For studies involving mixed HCT populations (malignant and non-malignant indications), we will include the study if more than 50% of the patients received HCT for malignant conditions. We will only include studies with a sample size of > 10 patients.

We will exclude studies restricted to adult patients (> 21 years old), premature neonates (≤ 36 weeks’ gestation), and mixed populations where data from pediatric cancer patients cannot be extracted separately. We will also exclude studies of non-malignant (benign) tumors and studies including children undergoing HCT where more than 50% are for non-malignant disease to reduce heterogeneity and to maintain focus on defining organ dysfunction within the oncologic context, which differs in its clinical course, risk profile, and therapeutic exposures.

### Definition of organ dysfunction and study scope

This study focuses on acute single-organ and multiorgan dysfunction (MOD) in pediatric oncology and post-HCT populations. The organ systems of interest include: respiratory, cardiovascular, neurologic, renal, hepatic, endocrine, endothelial, coagulation, immune, hematologic, and gastroenterology, as well as multiorgan dysfunction, defined as the concurrent dysfunction of two or more organ systems. For the purposes of study inclusion and search consistency, the core panel of experts defined organ toxicity as acute organ dysfunction or failure occurring within 12 months of cancer-directed therapy or hematopoietic cell transplantation (HCT), treated in a hospital setting (PICU, wards, or inpatient), and associated with at least one clinical outcome of interest, such as increased mortality, significant morbidity, prolonged hospitalization, or need for life-sustaining interventions.

Studies focusing solely on chronic organ dysfunction or dysfunction identified in ambulatory settings (e.g., outpatient clinics) are excluded, as we aim to characterize and adapt criteria relevant to acute, hospital-based deterioration in this high-risk population.

### Outcome and association

Included studies must report outcomes associated with organ dysfunction or failure, including mortality and morbidity (length of hospital stay, rehospitalization, reoperation, etc.), and analyze how specific organ dysfunction characteristics are statistically associated with these outcomes in the target patient populations. Studies that do not report specified outcomes or evaluate the relationship between organ dysfunction and outcomes are excluded. This focus aligns with our primary aim of identifying evidence-based organ dysfunction criteria linked to adverse outcomes, ensuring that any proposed adaptations to the original PODIUM criteria are rooted in prognostic significance and reflect the clinical trajectory of children with cancer or those undergoing HCT.

### Study design and timeframe

We will include original studies published since January 1, 2004, including prospective or retrospective cohort studies, surveillance studies, hospital database publications, cross-sectional studies, data from before-and-after studies, and registry data, regardless of language. The January 2004 cut-off was chosen to align with the methodology of the original PODIUM study and to provide a 20-year timeframe for data collection, which allows for a comprehensive assessment of evolving organ dysfunction definitions over time. While we recognize clinical practices have changed, especially in high-income countries, treatment protocols and diagnostic criteria used in earlier studies may still be relevant in low- and middle-income countries, supporting the global applicability of our adapted criteria.

We will exclude publications limited to abstracts, case reports with ≤ 10 patients, narrative reviews, surveys, study protocols, comments, editorials, letters, notes, conference materials, interventional trials, and any texts where full access to the article is unavailable.

### Data sources and search strategy

Our search strategy was collaboratively developed by multidisciplinary co-investigators, an academic librarian, and underwent feasibility testing. We employed a mix of controlled vocabulary terms, such as PubMed Medical Subject Headings (MeSH), and keywords derived from the original 2017 and updated 2020 PODIUM searches. Terms and search strings were curated based on team discussions, alignment with our research objectives, and recent updates to controlled vocabularies and database search algorithms. This strategy encompasses concepts for oncology, HCT/CAR, pediatric patients, multiorgan dysfunction, association analysis, and outcomes, resulting in a highly comprehensive and targeted modular search. To ensure capturing publications focused on the pediatric population, we limited results based on a comprehensive set of subject headings and keywords for “children” and “youth”, because there is no consistent or replicable way to limit search results based on years of age in literature databases. Specific years of age were applied to studies in the screening process. Organ toxicity will be captured using terms and concepts that refer to acute dysfunction, failure, or injury in specific organ systems. We will include search terms for both general and organ-specific toxicities (e.g., hepatotoxicity, nephrotoxicity, neurotoxicity, cardiotoxicity), as well as broader terms such as organ dysfunction, organ failure, and multiorgan dysfunction syndrome (MODS). Importantly, we will not rely solely on the term “toxicity” as it is often underused or inconsistently applied in clinical literature. Instead, we will use a combination of Controlled vocabulary (e.g., MeSH terms like Organ Dysfunction Scores, Organ Failure, Treatment-Related Adverse Events). Keywords referring to both clinical syndromes (e.g., acute kidney injury, sinusoidal obstruction syndrome, cytokine release syndrome) and pathophysiological terms indicating toxicity or damage. To ensure comprehensive inclusion, these terms will be paired with population filters (pediatrics, oncology, HCT) and outcome-related terms (e.g., mortality, prognosis, ICU admission). (The full description of the search terms is found in **Supplementary 1**).

The search results will be translated across databases and compiled into a Data Set (Reference Manager). In the Reference Manager, we will perform full-text searches for each organ system, enabling us to capture precise and comprehensive data from both abstracts and full-text articles. Each organ-specific search subset will then be uploaded individually into Covidence (23), a web-based platform designed to streamline systematic reviews, allowing us to assess overlaps and relationships between the different organ systems. This strategy will be applied to 11 organ-specific reviews (respiratory, cardiovascular, neurology, renal, hepatic, endocrine, endothelial, coagulation, immune, hematology, and gastroenterology) and one multisystem organ dysfunction review of scoring systems used to predict outcomes in children with cancer.

### Study selection and screening process

Titles retrieved from the search will be uploaded to Covidence for systematic review processes, including screening, full-text uploads, and conflict resolution. Citations will be assessed for eligibility based on title, abstract, and full text.

For each systematic review, a diverse group of experts in pediatric oncology, critical care, HCT, infectious disease, and relevant subspecialties were invited to join organ-specific Working Groups. To ensure consistency and accuracy, Working Group members will participate in a training session covering how to use Covidence software, study inclusion and exclusion criteria, and will complete a training set of 10 sample publications (titles and abstracts) before commencing abstract screening. The training set, prepared by the co-investigators, will intentionally include 3–5 true positives. Members of each Working Group will independently screen the training set and discuss their decisions during a group meeting to achieve alignment.

**Figure 2:**
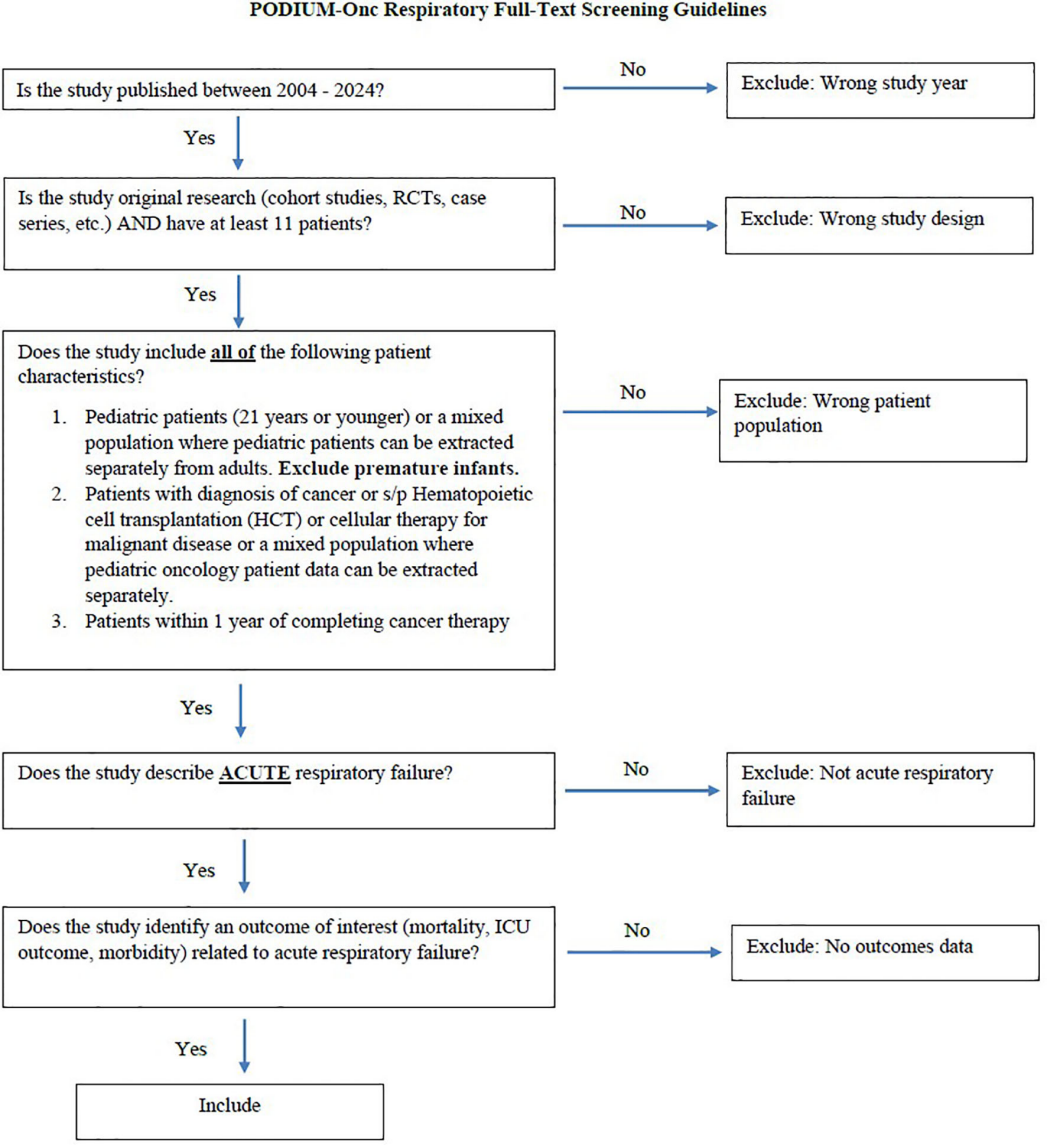
Full-text screening guide for Respiratory Failure; each-specific review will have a similar adapted guide.

For the main screening phase, per Cochrane recommendations in Covidence (24), each title and abstract will be independently reviewed by two Working Group members using predefined eligibility criteria. Titles rejected by both reviewers will be excluded, while those accepted by both will proceed to full-text screening. In cases of disagreement, a third reviewer will resolve the conflict. Each full-text article will be reviewed by two members of the Working Group to assess eligibility for inclusion in the final set for data extraction. Any conflicts during screening or assessment will be resolved by a third member of the Support Group using Covidence’s conflict resolution feature. For full texts with exclusion criteria, a reason for exclusion will be recorded (e.g., wrong study year; wrong study design (review, case series ≤ 10 patients); Wrong patient population (adults, not cancer patients); not acute organ dysfunction; no outcomes data; full text not found; duplicate publication). **Figure 2** provides a sample full-text screening guide for respiratory failure, and each organ-specific review will have a similar adapted guide.

Citations identified during title and abstract screening will be excluded if the full text cannot be retrieved after the following steps: searching journal subscriptions at two or more academic institutions, conducting a general web-based search using Google, submitting Interlibrary Loan requests to at least two academic institutions, and directly emailing the corresponding author or editor for the article. Publications with data from more than one country (e.g., global prevalence studies) will be included if they meet the inclusion criteria. For studies published in languages other than English, a bilingual member of the study team will assist in determining inclusion or exclusion.

### Data management and extraction

Data management in a large project is crucial. The expected volume of more than 100,000 articles to process presents practical management issues. Multiple organ systems may be addressed in one article, which increases the complexity of retrieving and sorting articles before dissemination to each team. A data management plan (**Supplementary 2**) was developed to establish viable details of document collection, organization, and short-term storage. Constructing the plan will help the team define procedures like file naming conventions, metadata variables, and assigning responsibilities at each stage of the process, thus addressing legal issues of sponsorship requirements and copyright.

Data from all eligible full-text articles will be extracted by one Working Group member and independently verified by a second member, and entered into REDCap, a secure web-based application designed for electronic data capture (25). During the screening phase, data extraction conflicts will be resolved by a third Working Group member. Data extraction will include information related to study characteristics (i.e., title, authors, year of publication, date of enrollment, language, journal, study design, sample size, inclusion/exclusion criteria, data source), hospital setting (general PICU, oncology PICU, oncology wards, emergency room and operating room), specific definitions of organ failure, outcomes (mortality, functional outcomes/residual morbidity, organ-specific outcomes, outcomes related to MODS, other patient-centered outcomes), and mitigating factors (factors or diagnoses leading that affect outcomes in the setting of organ failure). The data extraction form will be adapted from the original PODIUM study. Studies published in languages other than English will have data extracted by a bilingual member of the study team.

### Risk of bias in individual studies

The Quality in Prognosis Studies (QUIPS) (26) tool for prognostic factors studies will be used to assess risk of bias in the included studies. The following domains will be evaluated: study participation, study attrition, prognostic factor measurement, outcome measurement, potential confounding factors, and appropriateness of statistical analysis and reporting. Each of the 6 domains will be rated as having low, moderate, or high risk of bias. Two team members will independently assess the risk of bias, and any conflicts will be resolved by discussion or by a third member of the Working Group if consensus cannot be reached. The full QUIPS 6-domain questionnaire is provided in **Supplementary 3**.

### Data synthesis and analysis

Data will be analyzed for each systematic review (11 organ-specific reviews and 1 MODS search) to synthesize information on risk factors and definitions of organ dysfunction associated with poor outcomes. Data synthesis will include [1] quantitative analysis of the number of participants/episodes and strength of the relationship between given risk factor/organ dysfunction definition and outcome, and [2] narrative synthesis to identify mitigating factors impacting outcomes with organ dysfunction in children with cancer. Given the anticipated heterogeneity in study design and analytic techniques, a formal meta-analysis is not planned, and formal subgroup analysis will not be possible. However, where the literature allows, we will explore differences across patient populations, countries, study settings, study languages, study designs, types of data collection (prospective vs. retrospective), data sources (chart review, prospective data collection, electronic medical record [EMR] query, or registry), participating sites (single-center vs. multicenter), age categories (neonates: 0–30 days; infants: 31 days to <1 year; children: 1 to <12 years; adolescents: 12 to <18 years; young adults: 18 to 21 years), reason for HCT (malignant, mixed malignant and non-malignant), and type of HCT or cellular therapy (autologous HCT, allogeneic HCT, or CAR-T). These subgroup considerations have been incorporated into our Case Report Form (CRF) (**Supplementary 3**).

To address discrepancies in the evidence base, as was done in the original PODIUM study, this narrative synthesis will inform a modified Delphi process during the consensus-building phase. This process will reconcile divergent findings and inform the development of the adapted PODIUM-Onc criteria. The Delphi process will be conducted in accordance with the Conducting and REporting DElphi Studies (CREDES) guidelines to ensure methodological rigor, transparency, and robust stakeholder engagement (27).

## Anticipated results

Through these linked systematic reviews, we expect to identify characteristics of organ dysfunction associated with clinically important outcomes among children with cancer. Findings from these reviews will inform a planned expert modified consensus process to systematically adapt the existing PODIUM criteria for use in these unique and high-risk patients. Ultimately, this work will be used to standardize the identification and characterization of organ dysfunction in children with cancer in clinical care and research. This work will also establish a methodology for how to adapt PODIUM criteria for other populations. We anticipate that for some organ systems, this review will identify limited data on dysfunction criteria; this will serve to highlight critical gaps in the current literature and guide recommendations for future research efforts.

## Discussion

Children with cancer are a unique patient population requiring unified, systematic, and tailored definitions for single and multiorgan dysfunction to allow for research and informed clinical care. The proposed study will address this need by synthesizing data needed to inform adaptation of the PODIUM organ failure criteria to accurately identify and further study organ dysfunction in this vulnerable patient population. Globally, approximately 43% of childhood cancer cases were undiagnosed, with substantial regional variation, ranging from 3% in high-income countries (HICs) to 57% in low- and middle-income countries (LMICs), highlighting both inequities in access to care and limitations in available data. The adapted PODIUM-Onc criteria aim to provide a framework applicable across settings to support equitable advances in pediatric oncology critical care, reduce disparities in outcome measurement, and improve survival worldwide (28).

### Study strengths

This proposed study has several notable strengths. Our work leverages the methodology used to develop the original PODIUM criteria. The search strategy, data extraction, and bias assessment tools all build off the original study and are adapted to account for the unique pathophysiology, etiology, and outcomes of organ failure among children with cancer. Our study leadership team and Working Groups intentionally include investigators who contributed to the development of the original criteria.

In designing this study, we have also addressed some of the limitations of the original study. Our search strategy specifically focuses on terms unique to our target population, which were iteratively designed by our expert team. We will develop a comprehensive Data Set (Reference Manager), enabling full-text searches for organ failure terms—a necessary step given that much of the organ failure literature in children with cancer discusses MODS, rather than single-organ failure, and therefore would not be captured in a traditional search strategy. This review is planned as a one-time systematic review; however, the methodology has been designed to allow for straightforward updating in the future as new evidence becomes available. Furthermore, we will evaluate the applicability of the adapted PODIUM-Onc criteria to ensure that the resulting framework has a real-world impact and can meaningfully improve care for children with cancer.

Our diverse Working Group team intentionally includes investigators from a variety of specialties, disciplines, countries, and practice settings to provide comprehensive expertise on organ failure in this patient population. Importantly, the group also includes survivors of pediatric cancer, whose lived experiences bring valuable perspectives to the interpretation of findings and the development of organ dysfunction criteria relevant to this community.

We aim to comprehensively assess the global literature on organ dysfunction in pediatric cancer. To achieve this goal, we have not included any language filters in this search. Our global collaborative Working Group team includes investigators fluent in English, Spanish, Portuguese, German, Polish, Chinese, French, Turkish, Arabic, Russian, Italian, Dutch, Korean, and Urdu, among others. If a study is identified in a language not spoken by our primary study team, we will leverage the St. Jude Global Critical Care Program network of over 1300 clinicians from 80 countries to aid in screening and, if needed, data extraction. Through this approach, we are confident in our ability to rigorously evaluate all studies published on these topics in the current literature, regardless of language (29).

Additionally, we recognize this proposed work requires a large investigative team. As described, we have already assembled 104 collaborators (14 Core Team and 90 Working Group Members). More investigators will be invited as needed to ensure a comprehensive expert team to rigorously perform all systematic reviews.

### Potential challenges and proposed solutions

While the field of pediatric onco-critical care has experienced significant growth over the past decade, the proposed approach may encounter some limitations. First, there may be limited literature available for certain organ systems in children with cancer and post-HCT, as well as in LMIC settings, which will likely be underrepresented in the review (30). This underrepresentation could limit the generalizability of our findings to global populations. In such cases, we will report available data and describe needed areas of future work.

Second, children receiving HCT for malignancy often undergo more intensive conditioning regimens, have prior exposure to chemotherapy or radiation, and face unique oncologic complications influencing both the risk and trajectory of organ dysfunction. While many organ toxicities following HCT can occur across both malignant and non-malignant indications, we have intentionally limited our population to children with malignancy, including those who received HCT or cellular therapy for cancer indications. This decision was made to reduce heterogeneity and ensure the clinical relevance of adapted criteria specifically for children with cancer. We recognize the overlap in toxicities between malignant and non-malignant HCT populations and acknowledge that the resulting definitions may not fully apply to children undergoing HCT for non-malignant diseases. Nevertheless, we hope the criteria developed through this work will serve as a foundation to inform future efforts to adapt organ dysfunction criteria tailored to the non-malignant HCT population.

Third, a formal meta-analysis is not planned due to the anticipated heterogeneity across eligible studies. This heterogeneity includes variation in study design (retrospective vs prospective, single- vs multicenter, registry-based vs institutional), differences in the definitions of organ dysfunction, inconsistent reporting of outcomes (mortality, ICU admission, length of stay, quality of life, etc.), variability in underlying patient populations (different malignancies, prior therapies, transplant timing, and comorbidities), and additional heterogeneity anticipated between studies conducted in low- and middle-income versus high-income countries due to differences in healthcare infrastructure, diagnostic practices, supportive care resources, and treatment protocols. Additionally, the evolving definitions of organ dysfunction and the changes in treatment practices over time might add to the complexity. Pooling such heterogeneous data would not yield valid or interpretable summary estimates. Instead, we will conduct a structured narrative synthesis to transparently summarize the range of definitions and risk factors reported, describe associations with outcomes, and highlight areas of convergence and divergence across studies.

Finally, there is a risk of publication bias, as studies with significant or positive findings are more likely to be published than those with negative or inconclusive results.

## Conclusion

By systematically reviewing and analyzing organ-specific factors associated with patient outcomes, we aim to create consensus criteria that are clinically relevant for pediatric oncology patients and HCT patients to support future research in this vulnerable patient population.
