# Supplementary material for: Developing organ dysfunction diagnostic criteria for children with cancer and post-hematopoietic cell transplantation: protocol of systematic review
Source: Front Oncol. 2025 Oct 15;15:1591263. doi: 10.3389/fonc.2025.1591263 (PMC12568018; doi:10.3389/fonc.2025.1591263)
Supplement: Supplementary file 1 [file DataSheet1.docx]

***Supplementary Material***

**Developing Organ Dysfunction Diagnostic Criteria for Children with Cancer and Post-Hematopoietic Cell Transplantation: Protocol of Systematic Review**

| **Appendix** | **Page** |
| --- | --- |
| Supplement 1.  Search Strategy | 2 |
| Supplement 2.  Data Management Plan | 13 |
| Supplement 3.  PODIUM-Onc Case Report Form | 15 |

**Supplement 1.**  **Search Strategy**

**MODS**

"Multiple Organ Failure"[Mesh] OR "Organ Dysfunction Scores"[Mesh] OR "MODS"[tw] OR "NPMODS"[tw] OR "PELOD-2"[tw] OR "PELOD 2"[tw] OR "P-MODS"[tw] OR "P MODS"[tw] OR "OFI"[tw] OR "pSOFA"[tw] OR "p-SOFA"[tw] OR "p SOFA"[tw] OR "PIMs"[tw] OR "organ failure*"[tw] OR "failed organ*"[tw] OR "failing organs*"[tw] OR "organ dysfunction*"[tw] OR "organ disfunction*"[tw] OR "dysfunctioning organ*"[tw] OR "septic shock*"[tw]

**Onc OR BMT**

"neoplasms"[MeSH Terms:noexp] OR "Bone Marrow Transplantation"[MeSH Terms] OR "a m l*"[tiab] OR "AML"[tiab] OR "cancer*"[tiab] OR "carcinom*"[tiab] OR "hamartom*"[tiab] OR "hepatoblastom*"[tiab] OR "leiomyom*"[tiab] OR "leukaemi*"[tiab] OR "leukemi*"[tiab] OR "lymphom*"[tiab] OR "malignan*"[tiab] OR "medulloblastom*"[tiab] OR "melanom*"[tiab] OR "metasta*"[tiab] OR "myoma*"[tiab] OR "nephroblastom*"[tiab] OR "neuroblastom*"[tiab] OR "oncolog*"[tiab] OR "osteosarcom*"[tiab] OR "retinoblastom*"[tiab] OR "rhabdomyom*"[tiab] OR "rhabdomyosarcom*"[tiab] OR "sarcom*"[tiab] OR "tumor*"[tiab] OR "tumour*"[tiab] OR "HSCT"[tiab] OR "BMT"[tiab] OR "b m t*"[tiab] OR "HCT"[tiab] OR "H C T*"[tiab] OR ((transplant*[tiab]) AND ("bone marrow*"[tiab] OR "hematopoie*"[tiab] OR "haematopoie*"[tiab] OR "stem cell*"[tiab] OR "allogen*"[tiab]))

**Peds**

"Child"[MeSH Terms] OR "Infant"[MeSH Terms] OR "Adolescent"[MeSH Terms] OR "Minors"[MeSH Terms] OR "Pediatrics"[MeSH Terms] OR "Child Health Services"[MeSH Terms] OR "Infant"[tiab] OR "infants"[tiab] OR "infancy"[tiab] OR "newborn"[tiab] OR "newborns"[tiab] OR "neonatal"[tiab] OR "neonate"[tiab] OR "neonates"[tiab] OR "baby"[tiab] OR "babies"[tiab] OR "preterm"[tiab] OR "prematurity"[tiab] OR "toddler"[tiab] OR "toddlers"[tiab] OR "boy"[tiab] OR "boys"[tiab] OR "boyhood"[tiab] OR "girl"[tiab] OR "girls"[tiab] OR "girlhood"[tiab] OR "kid"[tiab] OR "kids"[tiab] OR "Child"[tiab] OR "childhood"[tiab] OR "children"[tiab] OR "preadolescent"[tiab] OR "preadolescents"[tiab] OR "preadolescence"[tiab] OR "Adolescent"[tiab] OR "adolescents"[tiab] OR "adolescence"[tiab] OR "juvenile"[tiab] OR "juveniles"[tiab] OR "youth"[tiab] OR "youths"[tiab] OR "teen"[tiab] OR "teens"[tiab] OR "teenager"[tiab] OR "teenagers"[tiab] OR "teenaged"[tiab] OR "pubescent"[tiab] OR "pubescence"[tiab] OR "prepubescent"[tiab] OR "prepubescence"[tiab] OR "pediatric"[tiab] OR "Pediatrics"[tiab] OR "paediatric"[tiab] OR "paediatrics"[tiab] OR "minor"[tiab] OR "Minors"[tiab] OR "young people"[tiab] OR "young person"[tiab] OR "kindergarten"[tiab] OR "preschool"[tiab] OR "high school"[tiab] OR "high schooler*"[tiab] OR "junior high"[tiab]

**Outcomes AND Precision**

("Treatment Outcome"[MeSH Terms:noexp] OR "Patient Outcome Assessment"[MeSH Terms] OR "Critical Care Outcomes"[MeSH Terms] OR "outcome*"[tiab] OR "Trauma and Stressor Related Disorders"[MeSH Terms] OR "Trauma and Stressor Related Disorders"[MeSH Terms:noexp] OR "stress disorders, traumatic"[MeSH Terms] OR "Depression"[MeSH Terms] OR "Depressive Disorder"[MeSH Terms] OR "Disabled Children"[MeSH Terms] OR "Stroke"[MeSH Terms] OR "Seizures"[MeSH Terms:noexp] OR "PTSD"[tiab] OR "stress disorder*"[tiab] OR "Depression"[tiab] OR "depressed"[tiab] OR "depressive"[tiab] OR "depressing"[tiab] OR "trauma*"[tiab] OR "mental health"[tiab] OR "mentally healthy"[tiab] OR "mental illness*"[tiab] OR "mentally ill"[tiab] OR "disability"[tiab] OR "disabilities"[tiab] OR "disable*"[tiab] OR "disabling"[tiab] OR "neurocognitive"[tiab] OR "neuro-cognitive"[tiab] OR "neuro-cognitive"[tiab] OR "neurocognition"[tiab] OR "neurodevelopmental"[tiab] OR "neuro-developmental"[tiab] OR "neuro-developmental"[tiab] OR "neurodevelopment"[tiab] OR "neuro-development"[tiab] OR "neuro-development"[tiab] OR "neurologic"[tiab] OR "neurological"[tiab] OR "cognitive"[tiab] OR "life support"[tiab] OR "mechanical support"[tiab] OR "mechanically supported"[tiab] OR "mechanical circulatory support"[tiab] OR "inotrope dependency"[tiab] OR "inotrope dependent*"[tiab] OR "assistive device*"[tiab] OR "assist device*"[tiab] OR "LVAD"[tiab] OR "VFD"[tiab] OR "extracorporeal membrane oxygenation"[MeSH Terms] OR "extracorporeal membrane oxygen*"[tiab] OR "ECMO"[tiab] OR "Stroke"[tiab] OR "strokes"[tiab] OR "seizure"[tiab] OR "Seizures"[tiab] OR "Cost of Illness"[MeSH Terms] OR "Health Care Costs"[MeSH Terms] OR "Health Expenditures"[MeSH Terms] OR "healthcare cost*"[tiab] OR "health care cost*"[tiab] OR "health expenditure*"[tiab] OR "cost of illness*"[tiab] OR "Mortality"[MeSH Terms] OR "Death"[MeSH Terms] OR "Length of Stay"[MeSH Terms] OR "Quality of Life"[MeSH Terms] OR "Life Expectancy"[MeSH Terms] OR "Quality-Adjusted Life Years"[MeSH Terms] OR "Patient Readmission"[MeSH Terms] OR "Reoperation"[MeSH Terms] OR "Mortality"[tiab] OR "mortalities"[tiab] OR "morbidity"[tiab] OR "morbidities"[tiab] OR "moribund"[tiab] OR "Death"[tiab] OR "died"[tiab] OR "dying"[tiab] OR "fatal"[tiab] OR "fatality"[tiab] OR "Length of Stay"[tiab] OR "stay length"[tiab] OR "length of hospital stay"[tiab] OR "LOS"[tiab] OR "Quality of Life"[tiab] OR "life quality"[tiab] OR "HRQol"[tiab] OR "QoL"[tiab] OR "PedsQL"[tiab] OR "qualy*"[tiab] OR "life year*"[tiab] OR "healthy year*"[tiab] OR "longevity"[tiab] OR "duration of life"[tiab] OR "life expectanc*"[tiab] OR "life table*"[tiab] OR "quality of dying"[tiab] OR "quality of death"[tiab] OR "death quality"[tiab] OR "Tracheostomy"[MeSH Terms] OR "Tracheostomy"[tiab] OR "Gastrostomy"[MeSH Terms] OR "Gastrostomy"[tiab] OR "gastric tube*"[tiab] OR "readmit*"[tiab] OR "readmission*"[tiab] OR "rehospitalization*"[tiab] OR "rehospitaliz*"[tiab] OR "reoperate*"[tiab] OR "reoperation*"[tiab] OR "reoperating"[tiab] OR "revision surger*"[tiab] OR "surgical revision*"[tiab] OR "symptom improvement*"[tiab] OR "POPC"[tiab] OR "WeeFIM"[tiab] OR "PEDI"[tiab] OR "WISC"[Title/Abstract] OR "FSS"[tiab] OR "NPMODS"[tiab] OR "BSID"[tiab] OR "pedNiHSS"[tiab] OR "GOS"[tiab] OR "cerebral performance"[tiab] OR "overall performance categor*"[tiab] OR "functional status scale*"[tiab] OR "mullen scale*"[tiab] OR "wechsler intelligence scale*"[tiab] OR "bayley scale*"[tiab] OR "vineland adaptive behavior scale*"[tiab] OR "glasgow outcome score*"[tiab] OR "stroke scale*"[tiab] OR (("Waiting Lists"[MeSH Terms] OR "waiting list*"[tiab]) AND ("Transplantation"[MeSH Terms] OR "transplant*"[tiab]))) AND ("Sensitivity and Specificity"[MeSH Terms] OR "Predictive Value of Tests"[MeSH Terms] OR "Prognosis"[MeSH Terms:noexp] OR "models, statistical"[MeSH Terms] OR "Costs and Cost Analysis"[MeSH Terms] OR "Risk"[MeSH Terms:noexp] OR "False Negative Reactions"[MeSH Terms] OR "False Positive Reactions"[MeSH Terms] OR "sensitiv*"[tiab] OR "specificit*"[tiab] OR "predict*"[tiab] OR "validat*"[tiab] OR "validity"[tiab] OR "prognostic*"[tiab] OR "statistical model*"[tiab] OR "risk*"[tiab] OR "false negative*"[tiab] OR "false positive*"[tiab] OR "accurac*"[tiab] OR "accurate"[tiab] OR "precision"[tiab] OR "precise"[tiab])

**Respiratory**

"Acute Lung Injury"[mesh] OR "Acidosis, Respiratory"[mesh] OR "Respiratory Distress Syndrome"[mesh] OR "Respiratory Insufficiency"[mesh:noexp] OR "Pulmonary Edema"[Mesh] OR "Respiration, Artificial"[Mesh] OR "Continuous Positive Airway Pressure"[Mesh] OR "Bronchiolitis Obliterans"[Mesh] OR "Cytokine Release Syndrome"[Mesh] OR "acute lung injuries"[tiab] OR "acute lung injury"[tiab] OR "ahrf"[tiab] OR "ALI"[tiab] OR "lung dysfunction*"[tiab] OR "lung failure*"[tiab] OR "lung function*"[tiab] OR "lung edema*"[tiab] OR "lung oedema*"[tiab] OR "pulmonary dysfunction*"[tiab] OR "pulmonary failure*"[tiab] OR "pulmonary distress*"[tiab] OR "pulmonary function*"[tiab] OR "pulmonary edema*"[tiab] OR "pulmonary oedema*"[tiab] OR "respiratory acidosis"[tiab] OR "respiratory distress*"[tiab] OR "respiratory dysfunction"[tiab] OR "respiratory failure"[tiab] OR "respiratory insufficiency"[tiab] OR "respiratory function*" OR "respiratory rate*"[tiab] OR "shock lung*"[tiab] OR "shocked lung*"[tiab] OR "tachypn*"[tiab] OR "ards"[tiab] OR "ventilation"[tiab] OR "ventilator*"[tiab] OR "respiration*"[tiab] OR "continuous positive airway"[tiab] OR "CPAP"[tiab] OR "organizing pneumon*"[tiab] OR "COP"[tiab] OR "bronchiolitis Obliterans"[tiab] OR "BOS"[tiab] OR "BOOP"[tiab] OR "cytokine release syndrome"[tiab] OR (("Lung"[Mesh] OR lung[tiab] OR lungs[tiab] OR pulmon*[tiab]) AND ("Graft vs Host Disease"[Mesh] OR "graft versus host"[tiab] OR "graft vs host"[tiab] OR "GVHD"[tiab] OR "Endothelin-1"[Mesh] OR "von Willebrand factor"[Mesh] OR "plasminogen activator inhibitor-1"[Mesh] OR "high flow nasal cannula"[tiab] OR "PaO2 FiO2"[tiab] OR "SaO2 FiO2"[tiab] OR "SpO2 FiO2"[tiab] OR "oxygenation"[tiab] OR "oxygen saturation index"[tiab] OR "blood ph"[tiab] OR "arterial oxygen"[tiab] OR "PaO2"[tiab] OR "PCO2"[tiab] OR "dead space fraction*"[tiab] OR "brain natriuretic protein"[tiab] OR "BNP"[tiab] OR "vWF"[tiab] OR "von Willebrand factor"[tiab] OR "ICAM-1"[tiab] OR "endothelin*"[tiab] OR "plasminogen activator*"[tiab] OR "PAI-1"[tiab] OR "idiopathic pneumonia"[tiab] OR "sepsis" OR "septic"))

**Cardiac**

"Arrhythmias, Cardiac"[Mesh] OR "Cardiac Output, Low"[Mesh] OR "Cardiac Tamponade"[Mesh] OR "Cardiomyopathy, Dilated"[Mesh] OR "Cardiomyopathy, Restrictive"[Mesh] OR "Cardiomegaly"[Mesh] OR "Myocarditis"[Mesh] OR "Cardiotoxicity"[Mesh] OR "Heart Failure, Diastolic"[Mesh] OR "Heart Failure, Systolic"[Mesh] OR "Ventricular Dysfunction"[Mesh] OR "Pulmonary Arterial Hypertension"[Mesh] OR "Troponin"[Mesh] OR "Cytokine Release Syndrome"[Mesh] OR "heart failure*"[tiab] OR "heart dysfunction*"[tiab] OR "heart function*"[tiab] OR "dysfunctional heart*"[tiab] OR "cardiac failure*"[tiab] OR "cardiac dysfunction*"[tiab] OR "cardiac function*"[tiab] OR "cardiac output*"[tiab] OR "dysfunctional cardio*"[tiab] OR "cardiovascular failure*"[tiab] OR "cardiovascular dysfunction*"[tiab] OR "myocardial failure*"[tiab] OR "myocardial dysfunction*"[tiab] OR "myocardial function*"[tiab] OR "cardiogenic failure*"[tiab] OR "cardiogenic function"[tiab] OR "ventricular failure*"[tiab] OR "ventricular dysfunction*"[tiab] OR "ventricular function*"[tiab] OR "ventricle failure*"[tiab] OR "ventricle dysfunction*"[tiab] OR "ventricle function*"[tiab] OR "myocarditis"[tiab] OR "cardiomega*"[tiab] OR "cardiomyopath*"[tiab] OR "carditis"[tiab] OR "cardiotox*"[tiab] OR "troponin*"[tiab] OR "AV-O2"[tiab] OR "a v differ*"[tiab] OR "vasoactive-inotropic"[tiab] OR "inotropic score*"[tiab] OR "VIS"[tiab] OR "cytokine release syndrom*"[tiab] OR (("Heart"[Mesh] OR "Cardiovascular System"[Mesh] OR "Heart Ventricles"[Mesh] OR "Myocardium"[Mesh] OR "heart"[tiab] OR "cardiovascular*"[tiab] OR "ventricular"[tiab] OR "ventricle*"[tiab] OR "cardiogenic*"[tiab] OR "myocardia*"[tiab] OR "myocardium*"[tiab]) AND ("spectroscopy, near infrared"[Mesh] OR "Lactic Acid"[Mesh] OR "Acidosis"[Mesh] OR "Oxygen Saturation"[Mesh] OR "Ultrasonography"[Mesh] OR "ischem*"[tiab] OR "arrhythmi*"[tiab] OR "lactate"[tiab] OR "lactic acid"[tiab] OR "lactatemia"[tiab] OR "Acidosis"[tiab] OR "SvO2"[tiab] OR "SV-O2"[tiab] OR "oxygen saturation*"[tiab] OR "venous saturation*"[tiab] OR "brain natriuretic peptide*"[tiab] OR "BNP"[tiab] OR "near infrared spectroscopy"[tiab] OR "NIRS"[tiab] OR "ultrasound*"[tiab] OR "fractional shortening"[tiab] OR "ejection fraction*"[tiab] OR "cerebral oximetr*"[tiab] OR "rSO2"[tiab] OR "plasma biomarker*"[tiab] OR "hypertensi*"[tiab] OR "systolic"[tiab] OR "diastolic"[tiab] OR "arteriovenous oxygen differ*"[tiab]))

**Neurology**

"Brain Edema"[Mesh] OR "Delirium"[Mesh] OR "Hypoxia, Brain"[Mesh] OR "brain dead"[tiab] OR "brain death*"[tiab] OR "PSOM"[tiab] OR "pediatric stroke outcome measure*"[tiab] OR "STESS"[tiab] OR "status epilepticus severity score*"[tiab] OR "EMSE"[tiab] OR "delirious"[tiab] OR "delirium"[tiab] OR "CAPD"[tiab] OR "confusion method"[tiab] OR "confusion assessment method"[tiab] OR "pCAM-ICU"[tiab] OR "psCAM-ICU"[tiab] OR "CAM-ICU"[tiab] OR "P-CAM"[tiab] OR "ICANS"[tiab] OR "TIANS"[tiab] OR neurotoxic*[tiab] OR ("neurologic*"[tiab] AND "toxic*"[tiab]) OR ((failure[tiab] OR dysfunction[tiab] OR function*[tiab]) AND ("Stroke"[Mesh:NoExp] OR "Coma"[mh:noexp] OR "Persistent Vegetative State"[Mesh] OR "Polyneuropathies"[Mesh:NoExp] OR "Hydrocephalus"[Mesh] OR "Encephalitis"[Mesh] OR "Cerebral Infarction"[Mesh] OR "Intracranial Hemorrhages"[Mesh] OR "stroke"[tiab] OR "strokes"[tiab] OR "coma"[tiab] OR "comas"[tiab] OR "comatose"[tiab] OR "vegetative state*"[tiab] OR "polyneuropathy*"[tiab] OR "hydrocephal*"[tiab] OR "altered mental stat*"[tiab] OR "cerebrovascular accident*"[tiab] OR "encephali*"[tiab] OR "encephalopath*"[tiab] )) OR (("Brain"[Mesh] OR "Central Nervous System"[Mesh:NoExp] OR brain*[tiab] OR "central nervous system*"[tiab] OR cerebral*[tiab] OR cranial*[tiab] OR cranium*[tiab] OR intracranial*[tiab] OR "intra-cranial*"[tiab] OR "intra cranial*"[tiab] OR subarachnoid[tiab] OR cognition[tiab] OR cognitive[tiab]) AND ("Graft vs Host Disease"[Mesh] OR "Cytokine Release Syndrome"[Mesh] OR "Venous Thrombosis"[Mesh] OR "Vasculitis"[Mesh] OR "Meningitis"[Mesh] OR "graft versus host"[tiab] OR "graft vs host"[tiab] OR "GVHD"[tiab] OR infarct*[tiab] OR hemorrhag*[tiab] OR haemorrhag*[tiab] OR edema*[tiab] OR oedema*[tiab] OR impare*[tiab] OR impairment*[tiab] OR "ischemi*"[tiab] OR "ischaemi*"[tiab] OR "hypoxia*"[tiab] OR "thrombo*"[tiab] OR "vasculiti*"[tiab] OR "meningi*"[tiab] OR "meningoencephal*"[tiab] OR "cytokine release syndrome*"[tiab]))

**Hepatic**

"Liver Failure"[Mesh] OR "Hepatitis"[Mesh] OR "Hepatorenal Syndrome"[Mesh] OR "Hypertension, Portal"[Mesh] OR "Hepatopulmonary Syndrome"[Mesh] OR "Hepatic Infarction"[Mesh] OR "Jaundice"[Mesh] OR "Hyperbilirubinemia"[Mesh] OR "Cholestasis"[Mesh] OR "Chemical and Drug Induced Liver Injury"[Mesh] OR "Hepatic Veno-Occlusive Disease"[Mesh] OR "Hepatomegaly"[Mesh] OR "Esophageal and Gastric Varices"[Mesh] OR "Common Bile Duct Diseases"[Mesh] OR "liver failure*"[tiab] OR "liver dysfunction*"[tiab] OR "dysfunctional liver*"[tiab] OR "liver function*"[tiab] OR "liver injur*"[tiab] OR "liver insufficien*"[tiab] OR "liver support*"[tiab] OR "hepatic failure*"[tiab] OR "hepatic dysfunction*"[tiab] OR "hepatic function*"[tiab] OR "hepatic injur*"[tiab] OR "hepatic insufficien*"[tiab] OR "hepatic support*"[tiab] OR "hepatomeg*"[tiab] OR "hepatopulmonary syndrome"[tiab] OR "hepatorenal syndrome"[tiab] OR "portal hypertension"[tiab] OR "cardiac hepatopath*"[tiab] OR "cholestas*"[tiab] OR "hyperbilirubinemi*"[tiab] OR "LIU score*"[tiab] OR "CLIF SOFA"[tiab] OR "Child-Turcotte Pugh"[tiab] OR "Model for End-Stage Liver Disease"[tiab] OR "MELD"[tiab] OR "aspartate aminotransferase to platelet ratio index"[tiab] OR "APRI"[tiab] OR (("Liver"[Mesh] OR "Hepatic Duct, Common"[Mesh] OR "Bile Ducts, Intrahepatic"[Mesh] OR "hepatic*"[tiab] OR "hepato*"[tiab] OR "intrahepat*"[tiab] OR "extrahepat*"[tiab] OR "bile duct*"[tiab] OR "biliary"[tiab]) AND ("Hyperammonemia"[Mesh] OR "Fibrosis"[Mesh:NoExp] OR "Bilirubin"[Mesh] OR "Vitamin D-Binding Protein"[Mesh] OR "necrosis"[tiab] OR "necroses"[tiab] OR "necroti*"[tiab] OR "ammonia*"[tiab] OR "coagulation"[tiab] OR "coagulopath*"[tiab] OR "infarct*"[tiab] OR "hyperammonemi*"[tiab] OR "fibrosis"[tiab] OR "fibroses"[tiab] OR "veno-occlusi*"[tiab] OR "veno occlusi*"[tiab] OR "stiff*"[tiab] OR "pulmonary hypertension"[tiab] OR "fibroscan"[tiab] OR "steatos*"[tiab] OR "obstruct*"[tiab] OR "statis"[tiab] OR "Kings College Hospital Criteria"[tiab] OR "bilirubin"[tiab] OR "Factor V"[mh] OR "Factor V"[tiab] OR "factor five"[tiab] OR "AC globulin"[tiab] OR "factor Pi"[tiab] OR "proaccelerin"[tiab] OR "factor 5"[tiab] OR "Factor VII"[mh] OR "Factor VII"[tiab] OR "factor seven"[tiab] OR "proconvertin"[tiab] OR "factor 7"[tiab] OR "Factor VIII"[mh] OR "Factor VIII"[tiab] OR "thromboplastinogen"[tiab] OR "factor 8"[tiab] OR "hyatt c"[tiab] OR "vitamin D"[tiab] OR "hydroxycholecalciferol"[tiab] OR "hydroxyvitamin d"[tiab] OR "transcalciferin"[tiab] OR "actin free Gc globulin"[tiab] OR "Gc globulin"[tiab]))

**Renal**

"Acute Kidney Injury"[Mesh] OR "Cardio-Renal Syndrome"[Mesh] OR "Renal Replacement Therapy"[Mesh] OR "Diabetes Insipidus, Nephrogenic"[Mesh] OR "Hepatorenal Syndrome"[Mesh] OR "Acidosis, Renal Tubular"[Mesh] OR "Fanconi Syndrome"[Mesh] OR "Kidney Tubular Necrosis, Acute"[Mesh] OR "Renal Dialysis"[Mesh] OR "Hemofiltration"[Mesh] OR "Proteinuria"[Mesh] OR "Oliguria"[Mesh] OR "Anuria"[Mesh] OR "Fanconi Syndrome"[tiab] OR "proteinuri*"[tiab] OR "oliguria*"[tiab] OR "anuria*"[tiab] OR "hypervolemi*"[tiab] OR "fluid overload"[tiab] OR "hemofilter*"[tiab] OR "hemodiafiltr*"[tiab] OR "hemodialysis"[tiab] OR "peritoneal dialysis"[tiab] OR "nephrotox*"[tiab] OR "nephrocheck*"[tiab] OR "proteinuria*"[tiab] OR "pRIFLE"[tiab] OR "pediatric RIFLE"[tiab] OR "AKIN"[tiab] OR "AKI network"[tiab] OR "KDIGO"[tiab] OR "kidney disease improving global outcomes"[tiab] OR "CVVH"[tiab] OR "CVVHD"[tiab] OR "ARRT"[tiab] OR "CVVHDF"[tiab] OR "CRRT"[tiab] OR "PIRRT"[tiab] OR "cystatin c"[tiab] OR (("Kidney"[Mesh] OR "Nephrons"[Mesh] OR "kidney*"[tiab] OR "nephron*"[tiab] OR "renal*"[tiab] OR "renovascular"[tiab]) AND ("Uremia"[Mesh] OR "TMA"[tiab] OR "thrombotic microangiopath*"[tiab] OR "edema*"[tiab] OR "oedema*"[tiab] OR "uremi*"[tiab] OR "fluid retention"[tiab] OR "ischemi*"[tiab] OR "ischaemi*"[tiab] OR "dialysis"[tiab] OR "insufficien*"[tiab] OR "necro*"[tiab] OR "deficien*"[tiab] OR "dysfunction*"[tiab] OR "function*"[tiab] OR "failure*"[tiab] OR "support*"[tiab] OR "injur*"[tiab] OR "syndrome*"[tiab] OR "replacement*"[tiab] OR "angina"[tiab] OR "neutrophil gelatinase-associated lipocalin"[tiab] OR "NGAL"[tiab] OR "TIMP2"[tiab] OR "TIMP-2"[tiab] OR "IGFBP7"[tiab] OR "tissue inhibitor metalloproteinase-2"[tiab] OR "IGF-binding protein-7"[tiab] OR "creatinine"[tiab]))

**Immunology**

"Agammaglobulinemia"[Mesh] OR "Agranulocytosis"[Mesh] OR "Chemokines"[Mesh] OR "Cytokines"[Mesh] OR "Dysgammaglobulinemia"[Mesh] OR "Eosinophilia"[Mesh] OR "Graft Enhancement, Immunologic"[Mesh] OR "Graft vs Host Disease"[Mesh] OR "Hematopoietic Cell Growth Factors"[Mesh] OR "HLA Antigens"[Mesh] OR "Hypereosinophilic Syndrome"[Mesh] OR "Infectious Mononucleosis"[Mesh] OR "Inflammasomes"[Mesh] OR "Interferons"[Mesh] OR "Interleukin-2"[Mesh] OR "Interleukins"[Mesh] OR "Leukocytes, Mononuclear"[Mesh] OR "Leukocytosis"[Mesh] OR "Leukostasis"[Mesh] OR "Lymphohistiocytosis, Hemophagocytic"[Mesh] OROR "Macrophage Activation Syndrome"[Mesh] OR "Monokines"[Mesh] OR "myeloid-derived suppressor cells"[Mesh] OR "T-Lymphocytes, Regulatory"[Mesh] OR "Transplantation Conditioning"[Mesh] OR "lipopolysaccharides"[Mesh] OR "lymphocyte depletion"[Mesh] OR "agammaglobulinemi*"[tiab] OR "agranulocytos*"[tiab] OR "ALC"[tiab] OR "ANC"[tiab] OR "CAR HLH"[tiab] OR "CARHLH"[tiab] OR "CAR-HLH"[tiab] OR "cell growth factor*"[tiab] OR "chemokine*"[tiab] OR "chimeric antigen receptor*"[tiab] OR "CXCL 10"[tiab] OR "CXCL 11"[tiab] OR "CXCL 9"[tiab] OR "CXCL10"[tiab] OR "CXCL11"[tiab] OR "CXCL9"[tiab] OR "cytokine*"[tiab] OR "depleted lymphoid*"[tiab] OR "dysgammaglobulinemi*"[tiab] OR "engraftment*"[tiab] OR "eosinophili*"[tiab] OR "graft enhancement*"[tiab] OR "graft rejection*"[tiab] OR "graft versus host"[tiab] OR "graft vs host"[tiab] OR "GVHD"[tiab] OR "hemophagocytic lymphohistiocytosis"[tiab] OR "hemophagocytic syndrome"[tiab] OR "HLA *"[tiab] OR "HLA-*"[tiab] OR "HLH *"[tiab] OR "HLH-*"[tiab] OR "hypereosinophili*"[tiab] OR "hyperinflamm*"[tiab] OR "IL 1 a"[tiab] OR "IL 1 alpha"[tiab] OR "IL 1 b"[tiab] OR "IL 1 beta"[tiab] OR "IL 1"[tiab] OR "IL 10"[tiab] OR "IL 11"[tiab] OR "IL 12"[tiab] OR "IL 13"[tiab] OR "IL 15"[tiab] OR "IL 16"[tiab] OR "IL 17"[tiab] OR "IL 18"[tiab] OR "IL 18"[tiab] OR "IL 1a"[tiab] OR "IL 1alpha"[tiab] OR "IL 1b"[tiab] OR "IL 1beta"[tiab] OR "IL 2"[tiab] OR "IL 23"[tiab] OR "IL 27"[tiab] OR "IL 3"[tiab] OR "IL 33"[tiab] OR "IL 4"[tiab] OR "IL 5"[tiab] OR "IL 6"[tiab] OR "IL 7"[tiab] OR "IL 8"[tiab] OR "IL 9"[tiab] OR "IL-1 a"[tiab] OR "IL-1 alpha"[tiab] OR "IL-1 b"[tiab] OR "IL-1 beta"[tiab] OR "IL-1"[tiab] OR "IL-10"[tiab] OR "IL-11"[tiab] OR "IL-12"[tiab] OR "IL-13"[tiab] OR "IL-15"[tiab] OR "IL-16"[tiab] OR "IL-17"[tiab] OR "IL-18"[tiab] OR "IL-18"[tiab] OR "IL-2"[tiab] OR "IL-23"[tiab] OR "IL-27"[tiab] OR "IL-3"[tiab] OR "IL-33"[tiab] OR "IL-4"[tiab] OR "IL-5"[tiab] OR "IL-6"[tiab] OR "IL-7"[tiab] OR "IL-8"[tiab] OR "IL-9"[tiab] OR "immune paralysis"[tiab] OR "immune suppress*"[tiab] OR "immunoparalysis"[tiab] OR "immunosuppress*"[tiab] OR "INF gamma"[tiab] OR "inflammasome*"[tiab] OR "interferon*"[tiab] OR "interleukin*"[tiab] OR "leukocyte antigen*"[tiab] OR "leukocyte disorder*"[tiab] OR "leukocytosis"[tiab] OR "leukopeni*"[tiab] OR "leukostasi*"[tiab] OR "lipopolysaccharide*"[tiab] OR "LPS"[tiab] OR "lymphocyte count*"[tiab] OR "lymphocytopeni*"[tiab] OR "lymphoid depletion"[tiab] OR "lymphopeni*"[tiab] OR "macrophage activation syndrome"[tiab] OR "marrow storage"[tiab] OR "MDSC*"[tiab] OR "monokine*"[tiab] OR "mononuclear cell*"[tiab] OR "mononuclear leukocyte*"[tiab] OR "myeloid derived suppressor cell*"[tiab] OR "myeloid precursor*"[tiab] OR "myeloid-derived suppressor cell*"[tiab] OR "neutropeni*"[tiab] OR "neutrophil count*"[tiab] OR "NLRP 3"[tiab] OR "NLRP3"[tiab] OR "pre-engraft*"[tiab] OR "regulatory t cell*"[tiab] OR "regulatory t lymphocyte*"[tiab] OR "regulatory t-cell*"[tiab] OR "regulatory t-lymphocyte*"[tiab] OR "TNF *"[tiab] OR "TNF-*"[tiab] OR "transplantation conditioning*"[tiab] OR "TREG"[tiab] OR "tumor necrosis factor*"[tiab] OR (("immune"[tiab] OR "immunity"[tiab]) AND ("deficien*"[tiab] OR "dysfunction*"[tiab] OR "disorder*"[tiab] OR "suppress*"[tiab] OR "dysregulat*"[tiab]))

**GIT**

"Ileus"[Mesh] OR "Intestinal Perforation"[Mesh] OR "Esophageal Perforation"[Mesh] OR "Typhlitis"[Mesh] OR "Enterocolitis"[Mesh] OR "Enterocolitis, Neutropenic"[Mesh] OR "Colitis"[Mesh] OR "Colitis, Ischemic"[Mesh] OR "Gastrointestinal Hemorrhage"[Mesh] OR "Hematemesis"[Mesh] OR "Melena"[Mesh] OR "Gastritis"[Mesh] OR "Ileus*"[tiab] OR "typhlit*"[tiab] OR "enterocolit*"[tiab] OR "coliti*"[tiab] OR "hematemes*"[tiab] OR "hematochezia*"[tiab] OR "melena*"[tiab] OR "gastriti*"[tiab] OR (("Gastrointestinal Tract"[Mesh] OR "Gastrointestinal Microbiome"[Mesh] OR "gastric*"[tiab] OR "gastro*"[tiab] OR "intestine*"[tiab] OR "intestinal"[tiab] OR "ileum"[tiab] OR "ileocecal"[tiab] OR "jejunum"[tiab] OR "jujunal"[tiab] OR "duodenum"[tiab] OR "duodenal"[tiab] OR "esophagus"[tiab] OR "esophageal"[tiab] OR "esophagogastric"[tiab] OR "stomach"[tiab] OR "colon"[tiab] OR "rectum"[tiab] OR "rectal*"[tiab] OR "gut"[tiab] OR bowel*[tiab]) AND ("Graft vs Host Disease"[Mesh] OR "Dysbiosis"[Mesh] OR "Malnutrition"[Mesh] OR "Wasting Syndrome"[Mesh] OR "Proteobacteria"[Mesh] OR "Metabolomics"[Mesh] OR "graft versus host*"[tiab] OR "graft vs host*"[tiab] OR "dybios*"[tiab] OR "malnutrition"[tiab] OR "wasting"[tiab] OR "proteobacteria*"[tiab] OR "failure"[tiab] OR "dysfunction*"[tiab] OR "function*"[tiab] OR "pneumatosis"[tiab] OR "perforat*"[tiab] OR "thrombotic microangiopath*"[tiab] OR "transplant associated microangiopath*"[tiab] OR "hemorrhag*"[tiab] OR "haemorrhag*"[tiab] OR "bleed"[tiab] OR "bleeding"[tiab] OR "feeding"[tiab] OR "microbiome*"[tiab] OR "microbial metabolit*"[tiab] OR "metagenomic*"[tiab] OR "malnutrition"[tiab] OR "hypermetabolic*"[tiab] OR "catabolic*"[tiab] OR "metabolomic*"[tiab] OR "bladder pressure"[tiab] OR "intra-abdominal hypertens*"[tiab] OR "intraabdominal hypertens*"[tiab] OR "intra abdominal hypertens*"[tiab] OR "intra-abdominal pressure"[tiab] OR "intraabdominal pressure"[tiab] OR "intra abdominal pressure"[tiab]OR "abdominal compartment syndrome" [tiab] OR "nasogastric output"[tiab] OR "phosphate*"[tiab] OR "phosphorus"[tiab] OR "prealbumin"[tiab] OR "proalbumin"[tiab] OR "transthyretin"[tiab] OR "fibrinogen"[tiab] OR "triglycerides"[tiab] OR "low density lipoprotein*"[tiab] OR "low density lipoprotein*"[tiab] OR "LDL"[tiab] OR "neopterin"[tiab] OR "C reactive protein"[tiab] OR "interleukin-6"[tiab] OR "IL-6"[tiab] OR "hydroxyvitamin D"[tiab]))

**Endothelial**

"Angiopoietins"[Mesh] OR "angiopoietin-2"[Mesh] OR "angiopoietin-1"[Mesh] OR "Intercellular adhesion molecule-1"[Mesh] OR "vascular cell adhesion molecule-1"[Mesh] OR "protein c"[Mesh] OR "protein s"[Mesh] OR "e-selectin"[Mesh] OR "p-selectin"[Mesh] OR "nitric oxide"[Mesh] OR "complement activation"[Mesh] OR "complement pathway, classical"[Mesh] OR "complement pathway, alternative"[Mesh] OR "complement system proteins"[Mesh] OR "complement membrane attack complex"[Mesh] OR "angiotensins"[Mesh] OR "angiotensin ii"[Mesh] OR "glycocalyx"[Mesh] OR "nitric oxide synthase type iii"[Mesh] OR "cell derived microparticles"[Mesh] OR "neutrophils"[Mesh] OR "phosphatidylserines"[Mesh] OR "thromboplastin"[Mesh] OR "thrombomodulin"[Mesh] OR "antithrombin iii"[Mesh] OR "transendothelial and transepithelial migration"[Mesh] OR "lipoprotein associated coagulation inhibitor"[Supplementary Concept] OR "fibrin fragment d"[Supplementary Concept] OR "heparin*"[riab] OR "angiopoietin*"[tiab] OR "protein c"[tiab] OR "protein s"[tiab] OR "nitric oxide"[tiab] OR "e selectin"[tiab] OR "p selectin"[tiab] OR "PADGEM protein*"[tiab] OR "vascular cell adhesion molecule*"[tiab] OR "VCAM"[tiab] OR "intercellular adhesion molecule*"[tiab] OR "ICAM"[tiab] OR "endothelial leukocyte adhesion molecule*"[tiab] OR "ELAM"[tiab] OR "peripheral arterial tonometr*"[tiab] OR "flow mediated dilation*"[tiab] OR "reactive hyperemia index"[tiab] OR "RHI"[tiab] OR "complement activation*"[tiab] OR "complement pathway*"[tiab] "classical pathway*"[tiab] OR "alternative pathway*"[tiab] OR "complement activation*"[tiab] OR "complement cascade*"[tiab] OR "complement system*"[tiab] OR "complement membrane*"[tiab] OR "total complement"[tiab] OR "C5 b9"[tiab] OR "C5b 9"[tiab] OR "C5b9"[tiab] OR "CH50"[tiab] OR "CH 50"[tiab] OR "d dimer"[tiab] OR "angiotensin*"[tiab] OR "glycocalyx"[tiab] OR "NFkB"[tiab] OR "reactive oxidative species"[tiab] OR "eNOS"[tiab] OR "neutrophil*"[tiab] OR "tissue factor expression*"[tiab] OR "thrombomodulin*"[tiab] OR "antithrombin*"[tiab] OR "tissue factor pathway*"[tiab] OR "cytokines IL 6"[tiab] OR "IL1beta"[tiab] OR "IL1 beta"[tiab] OR "CXCL 9"[tiab] OR "CXCL9"[tiab] OR "CXCL 10"[tiab] OR "CXCL10"[tiab] OR "CXCL 11"[tiab] OR "CXCL11"[tiab] OR "thrombotic microangiopath*"[tiab] OR "TMA"[tiab] OR (("Endothelium"[Mesh] OR "endothel*"[tiab]) AND ("dysfunction*"[tiab] OR "function*"[tiab] OR "failure*"[tiab] OR "activation*"[tiab] OR "microparticle*"[tiab] OR "permeab*"[tiab]))

**Endocrine**

"Adrenal Insufficiency"[Mesh:NoExp] OR "Hyperglycemia"[Mesh] OR "thyroxine"[Mesh] OR "Hypothyroidism"[Mesh] OR "thyrotropin"[Mesh] OR "adrenocorticotropic hormone"[Mesh] OR "triiodothyronine"[MesH] OR "Hyponatremia"[Mesh] OR "Hypernatremia"[Mesh] OR "Hypercalcemia"[Mesh] OR "Hyperkalemia"[Mesh] OR "Hypernatremia"[Mesh] OR "Hypocalcemia"[Mesh] OR "Hypokalemia"[Mesh] OR "Hyponatremia"[Mesh] OR "Hypophosphatemia"[Mesh] OR "Hyperphosphatemia"[Mesh] OR "Diabetes Insipidus"[Mesh] OR "Cushing Syndrome"[Mesh] OR "Water-Electrolyte Imbalance"[Mesh:NoExp] OR "hyperglycemi*"[tiab] OR "thyroxine"[tiab] OR "thyroxin"[tiab] OR "triiodothyronine"[tiab] OR "liothyronine"[tiab] OR "TSH deficien*"[tiab] OR "hyperthyroid*"[tiab] OR "thyrotropin"[tiab] OR "thyroid stimulating hormone*"[tiab] OR "adrenocorticotrop*"[tiab] OR "corticotropin*"[tiab] OR "ACTH"[tiab] OR "hyponatremi*"[tiab] OR "hypernatremi*"[tiab] OR "hypercalcemi*"[tiab] OR "hyperkalemi*"[tiab] OR "hypernatremi*"[tiab] OR "hypocalcemi*"[tiab] OR "hypokalemi*"[tiab] OR "hyponatremi*"[tiab] OR "hypophosphatemi*"[tiab] OR "hyperphosphatemi*"[tiab] OR "tumor lysis syndrome"[tiab] OR "inappropriate adh syndrome"[tiab] OR "addison disease"[tiab] OR "adrenoleukodystroph*"[tiab] OR "hypoaldosteron*"[tiab] OR "waterhouse friderichsen syndrome"[tiab] OR "panhypopituitar*"[tiab] OR "diabetes insipidus"[tiab] OR "SIADH"[tiab] OR "vasopressin*"[tiab] OR "antidiuretic hormone*"[tiab] OR "ADH"[tiab] OR "AVP deficien*"[tiab] OR "cushing*"[tiab] OR "electrolyte imbalance*"[tiab] OR "electrolyte balance*"[tiab] OR (("Endocrine System"[Mesh] OR "Vasopressins"[Mesh] OR "endocrin*"[tiab] OR "enteroendocrine*"[tiab] OR "vasopressin*"[tiab] OR "pituitary"[tiab] OR "adrenal*"[tiab] OR "adreno*"[tiab] OR "thyroid*"[tiab] OR "gland*"[tiab]) AND ("dysfunction*"[tiab] OR "function*"[tiab] OR "insufficien*"[tiab] OR "hypofunction*"[tiab] OR "hyperfunction*"[tiab] OR "stress dos*"[tiab] OR "glucos*"[tiab] OR "blood sugar"[tiab] OR "glycemia"[tiab] OR "cortisol"[tiab]))

**Coagulation**

"activated protein c resistance"[Mesh] OR "ADAMTS13 Protein"[Mesh] OR "afibrinogenemia"[Mesh] OR "alpha-2-antiplasmin"[Mesh] OR "angiotensin-converting enzyme inhibitors"[Mesh] OR "Antifibrinolytic Agents"[Mesh] OR "antithrombin III deficiency"[Mesh] OR "blood coagulation disorders"[Mesh] OR "blood coagulation factors"[Mesh] OR "Blood coagulation tests"[Mesh] OR "Carboxypeptidase B2"[Mesh] OR "caspase inhibitors"[Mesh] OR "Cell-Free Nucleic Acids"[Mesh] OR "coagulation protein disorders"[Mesh] OR "cysteine proteinase inhibitors"[Mesh] OR "dipeptidyl-peptidase IV inhibitors"[Mesh] OR "ecchymosis"[Mesh] OR "Ehlers-Danlos syndrome"[Mesh] OR "Extracellular Vesicles"[Mesh] OR "factor V deficiency"[Mesh] OR "factor VII deficiency"[Mesh] OR "Factor X deficiency"[Mesh] OR "factor Xa inhibitors"[Mesh] OR "Factor XI deficiency"[Mesh] OR "Factor XII deficiency"[Mesh] OR "Factor XII"[Mesh] OR "Factor XIIa"[Mesh] OR "Factor XIII deficiency"[Mesh] OR "fibrin fibrinogen degradation products"[Mesh] OR "fibrinogen"[Mesh] OR "Fibrinolysis"[Mesh] OR "hemangioma, Cavernous"[Mesh] OR "Hemorrhage"[Mesh:NoExp] OR "Heparin"[Mesh] OR "heparin, low-molecular- weight"[Mesh] OR "Histones"[Mesh] OR "hypoprothrombinemias"[Mesh] OR "International Normalized Ratio"[Mesh] OR "Matrix metalloproteinase inhibitors"[Mesh] OR "Partial thromboplastin time"[Mesh] OR "Plasma Exchange"[Mesh] OR "plasminogen inactivators"[Mesh] OR "platelet count"[Mesh] OR "protease inhibitors"[Mesh] OR "Proteasome inhibitors"[Mesh] OR "protein c deficiency"[Mesh] OR "Protein C"[Mesh] OR "protein s deficiency"[Mesh] OR "prothrombin time"[Mesh] OR "pseudoxanthoma elasticum"[Mesh] OR "purpura"[Mesh] OR "Purpura, Hyperglobulinemic"[Mesh] OR "Purpura, Thrombocytopenic"[Mesh] OR "scurvy"[Mesh] OR "serine peptidase inhibitors, Kazal Type"[Mesh] OR "serine proteinase inhibitors"[Mesh] OR "serpins"[Mesh] OR "shwartzman phenomenon"[Mesh] OR "telangiectasia, Hereditary hemorrhagic"[Mesh] OR "thrombelastography"[Mesh] OR "Thrombin time"[Mesh] OR "Thromboinflammation"[Mesh] OR "thrombomodulin"[Mesh] OR "Thromboplastin"[Mesh] OR "Tissue Plasminogen Activator"[Mesh] OR "trypsin inhibitors"[Mesh] OR "urokinase-type plasminogen activator"[Mesh] OR "vitamin k deficiency"[Mesh] OR "whole blood coagulation time"[Mesh] OR "activated protein c resist*"[tiab] OR "ADAMTS 13 protein"[tiab] OR "ADAMTS13 protein"[tiab] OR "afibrinogenemi*"[tiab] OR "angiotensin converting enzyme inhibit*"[tiab] OR "antifibrinolytic agent*"[tiab] OR "antifibrinolytic agent*"[tiab] OR "antiplasmin*"[tiab] OR "antithrombin III deficiency"[tiab] OR "antithrombin*"[tiab] OR "antithrombin*"[tiab] OR "bleed*"[tiab] OR "carboxypeptidase*"[tiab] OR "caspase inhibit*"[tiab] OR "cell free dna"[tiab] OR "Cleaving protease"[tiab] OR "clot density"[tiab] OR "clot formation"[tiab] OR "clot lysis"[tiab] OR "clot structure"[tiab] OR "clotting disorder*"[tiab] OR "clotting factor*"[tiab] OR "clotting time*"[tiab] OR "coagulation disorder*"[tiab] OR "coagulation factor*"[tiab] OR "coagulation test*"[tiab] OR "coagulation time*"[tiab] OR "coagulopath*"[tiab] OR "cryoprecipitate*"[tiab] OR "cysteine proteinase inhibit*"[tiab] OR "d dimer*"[tiab] OR "DIC"[tiab] OR "dipeptidyl peptidase IV inhibit*"[tiab] OR "ecchymosis"[tiab] OR "ehlers danlos"[tiab] OR "endogenous thrombin "[tiab] OR "extracellular vesicle*"[tiab] OR "factor 10*"[tiab] OR "factor 12*"[tiab] OR "factor ten"[tiab] OR "factor twelve"[tiab] OR "factor X*"[tiab] OR "factor10*"[tiab] OR "factor12*"[tiab] OR "factorX*"[tiab] OR "FFP"[tiab] OR "Fibrin degradation"[tiab] OR "fibrin fragment*"[tiab] OR "fibrinogen"[tiab] OR "fibrinogenemi*"[tiab] OR "fibrinolysis"[tiab] OR "fresh frozen plasma"[tiab] OR "FX II*"[tiab] OR "FXII*"[tiab] OR "haemorrhag*"[tiab] OR "haemorrhagic disorder*"[tiab] OR "haemorrhagic disorder*"[tiab] OR "haemorrhagic telangiectasia"[tiab] OR "hemangioma*"[tiab] OR "hemorrhagic disorder*"[tiab] OR "hemorrhagic disorder*"[tiab] OR "hemorrhagic telangiectasia"[tiab] OR "heparin*"[tiab] OR "histone"[tiab] OR "hyperfibrinogenemi*"[tiab] OR "hyperfibrinolysis"[tiab] OR "hypocoagul*"[tiab] OR "hypofibrinogenemi*"[tiab] OR "hypoprothrombinemi*"[tiab] OR "immune thrombosis"[tiab] OR "INR"[tiab] OR "International Normalized Ratio*"[tiab] OR "intravascular coagulation"[tiab] OR "intravascular disorder*"[tiab] "hemorrhag*"[tiab] OR "LMWH"[tiab] OR "matrix metalloproteinase inhibit*"[tiab] OR "microparticle*"[tiab] OR "netosis"[tiab] OR "neutrophils extracellular trap*"[tiab] OR "PAI"[tiab] OR "plasma exchange"[tiab] OR "plasmin inhibit*"[tiab] OR "plasminogen activator*"[tiab] OR "plasminogen inactivat*"[tiab] OR "plasminogen inhibit*"[tiab] OR "platelet aggregomet*"[tiab] OR "platelet count*"[tiab] OR "protease inhibit*"[tiab] OR "proteasome inhibit*"[tiab] OR "protein c"[tiab] OR "prothrombin time*"[tiab] OR "pseudoxanthoma elasticum*"[tiab] OR "PTT"[tiab] OR "purpura"[tiab] OR "rendu osler weber disease"[tiab] OR "rotational thromboelastometr*"[tiab] OR "ROTEG"[tiab] OR "ROTEM"[tiab] OR "scurvy"[tiab] OR "serine peptidase inhibit*"[tiab] OR "serine proteinase inhibit*"[tiab] OR "serpin*"[tiab] OR "shwartzman phenomenon"[tiab] OR "TAFI"[tiab] OR "TEG"[tiab] OR "thrombelastograph*"[tiab] OR "thrombin clotting time*"[tiab] OR "thrombin generation"[tiab] OR "thrombin time*"[tiab] OR "thrombin-activatable fibrinolysis inhibitor"[tiab] OR "thrombo inflammat*"[tiab] OR "thromboelastogra*"[tiab] OR "thromboelastograph*"[tiab] OR "thromboinflammat*"[tiab] OR "thrombomodulin"[tiab] OR "thromboplastin time*"[tiab] OR "tissue factor"[tiab] OR "tPA"[tiab] OR "trypsin inhibit*"[tiab] OR "uPA"[tiab] OR "vasopeptidase inhibit*"[tiab] OR "von Willebrand "[tiab] OR "VWFCP"[tiab] OR “fibrinolysis”[tiab] OR (("antithrombins"[Mesh] OR "antithrombin*"[tiab] OR "factor"[tiab] OR "protein"[tiab] OR "vitamin"[tiab]) AND ("deficien*"[tiab])) OR (("Plasma"[Mesh] OR "Blood Coagulation"[Mesh] OR "Blood Platelets"[Mesh] OR "plasma"[tiab] OR "coagul*"[tiab] OR "platelet*"[tiab] OR "clot"[tiab] OR "clotting"[tiab]) AND ("disorder*"[tiab] OR "dysfunction*"[tiab]))

**Hematology**

"Anemia"[Mesh] OR "Thrombophilia"[Mesh] OR "Polycythemia"[Mesh] OR "Pancytopenia"[Mesh] OR "Cytopenia"[Mesh] OR "Thrombocytosis"[Mesh] OR "Transfusion Reaction"[Mesh] OR "Reticulocytosis"[Mesh] OR "Platelet Count"[Mesh] OR "Blood Cell Count"[Mesh] OR "Erythrocyte Indices"[Mesh] OR "Erythrocyte Count"[Mesh] OR "Hematocrit"[Mesh] OR "Hemoglobinometry"[Mesh] OR "Haptoglobins"[Mesh] OR "Hemolysis"[Mesh] OR "Hepcidins"[Mesh] OR "Transfusion-Related Acute Lung Injury"[Mesh] OR "anemia*"[tiab] OR "anaemia*"[tiab] OR "anemic*"[tiab] OR "anaemic*"[tiab] OR "thrombocyt*"[tiab] OR "thrombophil*"[tiab] OR "thrombastheni*"[tiab] OR "polycythemi*"[tiab] OR "erythrocyte*"[tiab] OR "pancytopeni*"[tiab] OR "cytopeni*"[tiab] OR "reticulocytos*"[tiab] OR "reticulocytopeni*"[tiab] OR "transfusion reaction*"[tiab] OR "platelet volume"[tiab] OR "platelet count*"[tiab] OR "blood cell count*"[tiab] OR "red cell count*"[tiab] "erythrocyte index"[tiab] OR "erythrocyte indices"[tiab] OR "erythrocyte count"[tiab] OR "hematocrit"[tiab] OR "hemoglobinometr*"[tiab] OR "hemoglobin determination"[tiab] OR "free hemoglobin"[tiab] OR "haptoglobins"[tiab] OR "haptoglobin"[tiab] OR "hemolysis"[tiab] OR "hepcidin*"[tiab] OR "prohepcidin*"[tiab] OR "sequestration"[tiab] OR "bone marrow failure*"[tiab] OR "platelet deficienc*"[tiab] OR "platelet storage pool deficienc*"[tiab] OR "plateletpheres*"[tiab] OR "blood cell transfusion*"[tiab] OR "blood transfusion*"[tiab] OR "graft failure*"[tiab] OR "blood disorder*"[tiab] OR "hemolytic disorder*"[tiab] OR "haemolytic disorder*"[tiab] OR "hematological disorder*"[tiab] OR "haematological disorder*"[tiab] OR "hematological dysfunction*"[tiab] OR "haematological dysfunction*"[tiab] OR "hemolytic function*"[tiab] OR "haemolytic function*"[tiab] OR "hematological function*"[tiab] OR "haematological function*"[tiab] OR "hematological failure*"[tiab] OR "haematological failure*"[tiab]

**Supplement 2.**  **Data Management Plan**
 
(DRAFT) Data Management Plan for PODIUM Onc Systematic Review (DRAFT)

1. Raw data will include search strategies, bibliographic records, and full text articles.
2. Search strategies for each organ system and a separate search for multiple organs (MOD) will be developed by the Systematic Review Librarian and the Core Team in Word and stored in a Google Docs shared space while in draft stage.  Once finalized, the Word document will be converted to PDF.  The master document will be held by the Systematic Review Librarian in the shared space.  After the project, the final document will be placed in a data repository. such as OSF or Harvard Dataverse.
3. Bibliographic records will be downloaded from multiple bibliographic databases in the RIS format and uploaded into Covidence by the Systematic Review Librarian for de-duplication and initial scanning by the Core Team.
4. A master database of full text articles and citations will be created and maintained by the Data Management Librarian for the duration of the study.  RIS files will be uploaded into an EndNote Library.  Each organ system search is expected to vary in size from 5,000 to 20,000 citations, an estimated 150,000 citations after de-duplication.  Separate EndNote libraries will be created for each search organ system.  A set of text files will be created that reflect the citations for each organ system and placed in a repository.
5. The MOD search will be treated differently.  Full text articles will be retrieved, scanned and sorted by the organ system mentioned in the articles.  A subset of bibliographic records will be added to each organ system EndNote Library.  This will introduce duplicates into the overall study but keep each organ system citation unique.  Any full text articles retrieved for the study will not be shared afterwards due to copyright.
6. Processed data will be created during the screening and analysis stages of the study.
7. Covidence will be used to manage the article screening and review of full text articles.
8. Data extraction will be managed in RedCap.  An electronic data collection form will be used by team members to extract data from articles.  A copy of the data extraction form (PDF) will be deposited in the chosen repository and a DOI will be submitted with any publications.
9. Analyzed data will be tables and figures created for publications about the project.  These may include PRISMA flow diagram, table of included studies, forest plots, etc. Text, images and figures will use Word, TIF, PNG or other format per publishers’ guidance.
10. Final data will include complete search strategies for all databases (PDF).  May include statistical code and data files (CSV or Excel) to comply with study sponsor requirements. All data will be licensed through the Creative Commons.
11. Documentation and Metadata will be a group of items deposited together in a data repository such as OSF or Harvard Dataverse in an open-source format.  These items will include:
12. “Readme” file with a list of team members and who are assigned to key roles in the project.  For example, the Principal Investigators and the Systematic Review Librarian will be responsible for documenting each stage of the review; different Core Team members will lead the creation and editing of publications and study decisions such as exclusion criteria, data encoding, etc.
13. Tools created for the study such as the data extraction form.
14. Search strategies for each organ system.
15. Bibliography of citations organized by organ system.
16. A DOI link to the group of items placed in the repository will be shared at publication.
17. File naming conventions and version control
18. File structure for each project, PODIUMOnc Organ System Block will contain three subfolders: PODIUMOnc Search Documents, PODIUMOnc Search Results and PODIUMOnc Translations Documents.  Search Documents will contain the original test searches named using the following convention: STATUS System, (ie., DRAFT Kidney_Renal, FINAL Kidney_Renal).  Translation Documents will be the final searches translated into the syntax of each database to be used.  These will also maintain the database results statistics.   Search Results will contain the actual downloads of electronic and manual searches.
19. Retrieval structure of downloads: Since these searches are large with thousands of records, downloads will be grouped and placed in a ZIP file.  Database limits force downloads in subsets which will be named to facilitate organization.  For example, PODIUMOnc_Brain_Embase_2009-2006_413.ris where Brain is the system, Embase is the database, 2009-2006 is the time span and 413 is the count of citations retrieved in this set.
20. Hand searches by the team members will be placed under the search results folder in separate files with the following naming structure: System_hand_search_# of items found (i.e., renal_hand_search_2.txt)
21. Storage and back-up will be managed by two groups in the team, the Data and Records Management Group which is comprised of the Systematic Review Librarian and the Data Management Librarian and the Manuscript Group which are the researchers.
22. The librarians are responsible for managing active documents, back-ups and minimizing risks to the study caused by technical failures.  The Systematic Review Librarian will provide oversite of the search, de-duplication, and retrieval of articles.  The Data Librarian will maintain a master and two back-ups of the RIS files, original PDFs, PDFs post OCR validation and EndNote libraries for each organ/organ system.  The original will sit on the organization’s network drive (T_drive).  Back-up 1 will sit on OneDrive.  Back-up 2 will sit on an external hard drive in the library.  Working copies will be used by the teams.  Upon completion of the study, one set of data will be converted to a non-proprietary format and placed in longer term storage at the organization for use in any potential updates.  Sharable items will be placed in an external repository and the remaining working copies will be deleted.
23. Writing, editing and managing manuscript version control is the responsibility of the Manuscript Group.  Working documents will be held in a shared Teams space.  The Principal Investigator will designate a responsible person to maintain the Teams space and working documents until publication of the study.

**Supplement 3. PODIUM-Onc Case Report Form**

Section 1: Introduction Questions

1. Record ID (free text):
2. First Author’s Last Name (free text):
3. Year of Publication (free text):
4. Name of first reviewer:
5. Name of second reviewer:
6. Please upload the full text of the article here (file upload):
7. If relevant, please upload the supplement to the full text of the article here (file upload):
8. If relevant, please upload the second supplement to the full text of the article here (file upload):
9. Should the article be excluded (multiple choice)? • Include • Exclude
10. Why should it be excluded (multiple choice)?

• Wrong publication year (<2004) • Wrong study design (systematic review, commentary, editorial, or case series <11 patients) • Wrong patient population (adults or non-malignancy) • Not acute organ dysfunction (≥1 yr after therapy or outpatient) • No outcomes data

1. Study Language (multiple choice):

• English • Arabic • Chinese • Croatian • Czech • French • Farsi • German • Hebrew • Japanese • Korean • Polish • Portuguese • Russian • Spanish • Turkish • Other (specify)

1. Language, other (free text):
2. Funding source (multiple choice):

• Government (e.g., FDA, NIH, CDC, CIHR) • NGO (e.g., scientific societies, hospital foundations) • Industry • None • Not reported • Other (specify)

1. Funding source, other (free text):
2. Study Design (multiple choice):

• Observational (cohort, case-control, cross-sectional) • Quasi-experimental (pre/post, historic control, etc.) • Interventional (RCT or other) • Not clarified/unknown • Other (specify)

1. Study Design, other (free text):
2. How was data collected (multiple choice): • Prospective • Retrospective • Not clarified/unknown
3. Number of participating sites (multiple choice): • Single center • Multiple centers • Not clarified/unknown
4. Number of centers (free text):
5. What country was the study conducted (check all that apply):

Don't use university of language to determine. However, if introduction/discussion leads you to believe that this study was done in a particular context - "We conducted this study in a German context" or something like this, then you can assume the country.

• United States • Australia • Brazil • Canada • China • France • Germany • Greece • India • Italy • Japan • Malaysia • Norway • Portugal • Qatar • Saudi Arabia • Spain • Taiwan • United Kingdom • United Arab Emirates • Vietnam • Not specified • Other (specify)

1. Study Conducted, other (free text):
2. Were the years of study data collection reported (multiple choice): • Yes • No
3. What is the first year data was collected (free text):
4. What is the last year data was collected (free text):
5. What is the Study Setting (check all that apply): • Multidisciplinary PICU • Oncology PICU • Adult ICU with pediatric patients • PICU of unknown composition • General ward outside ICU • Oncology ward outside ICU • Emergency room (DR) • Operating room (OR) • Not clarified/unknown • Other (specify)
6. Study setting, other (free text):
7. Data source(s) (check all that apply): • Chart review • Prospective data collection • EMR query • Registry • Not clarified/unknown • Other (specify)
8. Data source, other (free text):
9. Study inclusion criteria (check all that apply): • ICU admission • Cancer diagnosis • HCT or cellular therapy • Organ failure (single or MODS) • Sepsis/infection • ARDS/respiratory failure • Not clarified/unknown • Other (specify)
10. Inclusion criteria, other (free text):
11. Type of HCT or cellular therapy (select all that apply) [shown if C is selected in Q29]:
     • HCT-auto • HCT-allo • CAR-T • Not clarified/unknown • Other (specify)
12. Type of HCT, other (free text):
13. Reason for HCT (multiple choice) [shown if C is selected in Q29]:
     • All malignancy • Mixed malignant and non-malignant disease • Not clarified/unknown
14. Sample size in full paper (n) (free text):
15. Sample size in target population (children with cancer/post-HCT for malignancy) (n) (free text):
    Note: if all patients in the target population, this question should be the same as above
16. Level of the Sample Size (multiple choice):

• Patient • Hospital admission • ICU admission

1. Age category in target population (check all that apply):
   Note: This is inclusion/exclusion criteria for the study

• Neonates (0–30 days) • Infants (31 days to <1 year) • Children (1–<12 years) • Adolescents (12–<18 years) • Young adults (18–21 years) • Not clarified/unknown

1. Age details (check all that apply):
   Note: This is the patient population of the study

• Mean • Median • Youngest age included • Oldest age included

1. Mean (free text) [shown if A is selected in Q38]:
   (Note: just provide the mean, not standard deviation)
2. Median (free text) [shown if B is selected in Q38]:
   (Note: just provide the median, not the interquartile range)
3. Youngest Age Included (Age) (free text) [shown if C is selected in Q38]:
4. Oldest Age Included (Age) (free text) [shown if D is selected in Q38]:
5. Does this article pertain to organ dysfunction related to sepsis, septic shock, or specific type of infection (e.g. pneumonia, meningitis, blood stream infection, etc) (multiple choice):

• Yes, all patients • Yes, some patients • No patients • Unclear

1. PODIUM Organ Group (check all that apply):

• Respiratory • Cardiovascular • Neurologic • Renal • Hepatic • Heme/Immune • Endocrine • Endothelial • Coagulation • Gastro-intestinal (GIT) • MODS scoring system

Section 2-12: Organ Specific Extraction

Section 2: Respiratory

1. Resp: Number of patients included in respiratory assessment (free text):
2. Resp: Score/assessment tool under study (AKA risk factor) (select all that apply):

• Resp rate • ARDS • P/F ratio • S/F ratio • OI • OSI • Binasal cannula (low-flow O₂) • High-flow nasal cannula (HFNC) • Non-invasive ventilation (NIV: CPAP/BiPAP) • Invasive mechanical ventilation • HFOV • ECMO • Biomarkers • Other (specify)

1. Resp: Resp scoring assessment, other (free text):
2. Resp: Please describe any data/notes not already listed that you think are important for interpretation of the data (if there is no additional information that you wish to explain, please leave this question blank) (free text):

Section 3: Cardiac

1. Cards: Number of patients included in Cardiovascular assessment (free text):
2. Cards: Score/assessment tool under study (AKA risk factor) (select all that apply):

• Heart rate • Blood pressure • Heart rate variability • Core vs peripheral temperature • Lactate • Cardiac enzymes (e.g., troponin) • BNP • Cerebral oximetry • Somatic oximetry • Mixed venous saturation • Echocardiogram findings (e.g., EF, SF) • Need for vasoactive support • Cardiac severity score (e.g., VIS) • ECMO (VA) • Cardiac arrest (e.g., CPR) • Other (specify)

1. Cards: cards scoring assessment, other (free text):
2. Cards: Please describe any data/notes not already listed that you think are important for interpretation of the data (if there is no additional information that you wish to explain, please leave this question blank) (free text):

Section 4: Neurology

1. Neuro: Number of patients included in Neurologic assessment (free text):
2. Neuro: Score/assessment tool under study (AKA risk factor) (select all that apply):
    • AVPU • GCS • Pupillary reaction • EEG • Somatosensory evoked potentials (SSEP) • Delirium (e.g., CAPH, pCAM-ICU) • Radiologic findings (e.g., PRES) • Neuropathy • Functional scoring (e.g., Karnovsky) • Acute anatomic defect (e.g., hydrocephalus, thrombosis, hemorrhage) • Requirement of acute surgical intervention (e.g., shunt placement) • Other altered mental status measures • Other (specify)
3. Neuro: neuro scoring assessment, other (free text):
4. Neuro: Please describe any data/notes not already listed that you think are important for interpretation of the data (if there is no additional information that you wish to explain, please leave this question blank) (free text):

Section 5: Renal

1. Renal: Number of patients included in Renal assessment (free text):
2. Renal: Score/assessment tool under study (AKA risk factor) (select all that apply):

• Acute kidney injury score/definition (e.g., RIFLE, pRIFLE, AKIN, KDIGO) • Initiation of RRT • Urine biomarkers • Plasma biomarkers • Fluid overload • Urine output • eGFR • Serum creatinine • Estimated creatinine clearance (eCrCl) • Other (specify)

1. Renal: Renal scoring assessment, other (free text):
2. Renal: Please describe any data/notes not already listed that you think are important for interpretation of the data (if there is no additional information that you wish to explain, please leave this question blank) (free text):

Section 6: Hepatic

1. Hepatic: Number of patients included in Hepatic assessment (free text):
2. Hepatic: Score/assessment tool under study (AKA risk factor) (select all that apply):

• Liver failure definition or score (e.g., Kings College Hospital Criteria, Liver Injury Unit (LIU) • Score, CLIF-SOFA, Child-Turcotte Pugh, MELD, APRI, etc.) • Veno-occlusive disease (VOD) • Sinusoidal Obstructive Syndrome (SOS) • Liver Function Test, AST • Liver Function Test, ALT • Ammonia • Bilirubin • GGT • Radiologic findings (e.g., ultrasound doppler, elastography, etc)

• Other (please specify)

1. Hepatic: Hepatic scoring assessment, other (free text):
2. Hepatic: Please describe any data/notes not already listed that you think are important for interpretation of the data (if there is no additional information that you wish to explain, please leave this question blank) (free text):

Section 7: Heme-Immune

1. Heme/Immune: Number of patients included in Heme/Immune assessment (free text):
2. Heme/Immune: Score/assessment tool under study (AKA risk factor) (select all that apply):

• Platelet count/function (thrombocytopenia) • RBC indices (Hb, MCV, MCH, etc.) • Markers of hemolysis: haptoglobin • Markers of hemolysis: indirect bilirubin • Markers of hemolysis: LDH • Graft failure • Transfusions (RBC, platelets) • Transfusion-related complications (e.g., TRALI) • Serum/plasma cytokine concentration • Ferritin • Graft-versus-Host Disease • Lymphocyte counts (ALC, subsets) • Immunoglobulin level • Mononuclear cell count (ANC, neutropenia) • Hyperinflammation (e.g., CRS, IEC-HS, HLH; not ferritin alone) • Immunoparalysis • Other (specify)

1. Heme/Immune: Heme/Immune scoring assessment, other (free text):
2. Heme/Immune: Please describe any data/notes not already listed that you think are important for interpretation of the data (if there is no additional information that you wish to explain, please leave this question blank) (free text):

Section 8: Endocrine

1. Endocrine: Number of patients included in Endocrine assessment (free text):
2. Endocrine: Score/assessment tool under study (AKA risk factor) (select all that apply):

• Hypoglycemia • Hyperglycemia • Adrenal axis evaluation (e.g., cortisol) • Thyroid function evaluation • Hyponatremia (e.g., SIADH) • Hypernatremia (e.g., diabetes insipidus) • Tumor lysis syndrome • Hypokalemia • Hyperkalemia • Hypophosphatemia • Hyperphosphatemia • Hypocalcemia • Hypercalcemia • Other electrolyte imbalances • Other (specify)

1. Endocrine: Endocrine scoring assessment, other (free text):
2. Endocrine: Please describe any data/notes not already listed that you think are important for interpretation of the data (if there is no additional information that you wish to explain, please leave this question blank) (free text):

Section 9: Endothelial

1. Endothelial: Number of patients included in Endothelial assessment (free text):
2. Endothelial: Score/assessment tool under study (AKA risk factor) (select all that apply):

• Serum/plasma biomarkers (e.g., VCAM-1, ICAM-1, angiopoietin, selectin, D-dimer) • Reactive hyperemia index (RHI) • Flow-mediated dilation • Complement activation: CH50 • Complement activation: sC5b-9 • Complement activation: AH50 • Complement activation: Other • Thrombotic microangiopathy (TMA) • Other (specify)

1. Endothelial: Endothelial scoring assessment, other (free text):
2. Endothelial: Please describe any data/notes not already listed that you think are important for interpretation of the data (if there is no additional information that you wish to explain, please leave this question blank) (free text):

Section 10: Coagulation

1. Coagulation: Number of patients included in Coagulation assessment (free text):
2. Coagulation: Score/assessment tool under study (AKA risk factor) (select all that apply):

• Coagulation Time, PT • Coagulation Time, APTT • Coagulation Time, ACT • Coagulation Time, INR • Elevated D-dimer • DIC • Hemorrhage, bleeding • Thrombosis/Hypercoagulation • Transfusions (i.e. cryoprecipitate, fresh frozen plasma, PCC, etc.) • Coagulation Factor Concentration, Protein C • Coagulation Factor Concentration, Protein S • Coagulation Factor Concentration, Factor VII • Coagulation Factor Concentration, ADAMTS13 • Coagulation Factor Concentration, Antithrombin • Coagulation Factor Concentration, TEG • Coagulation Factor Concentration, ROTEM • Coagulation Factor Concentration, ROTEG • Coagulation Factor Concentration, Other • Other (please specify)

1. Coagulation: Coagulation scoring assessment, other (free text):
2. Coagulation: Please describe any data/notes not already listed that you think are important for interpretation of the data (if there is no additional information that you wish to explain, please leave this question blank) (free text):

Section 11: GIT

1. GIT: Number of patients included in Gastrointestinal assessment (free text):
2. GIT: Score/assessment tool under study (AKA risk factor) (select all that apply):

• Measures of GI perfusion (e.g., somatic/flank NIRS) • Bowel ischemia (bowel perforation or pneumatosis intestinalis or ischemia present on gross resection or imaging, GI sloughing) • Measures of intra-abdominal hypertension (e.g., bladder pressure) • Enterocyte function biomarkers (e.g., IFABP, LFABP, citrulline, etc) • Markers of acquired acute malnutrition (e.g., Ph, pre-albumin, etc) • Feeding intolerance (e.g., emesis, ileus, typhlitis, GI hemorrhage, diarrhea, gastric residual volume, etc) • Markers of microbiome health (e.g., gut microbial metabolites, loss of diversity, etc) • Obesity • Undernutrition • Albumin level • Pancreatitis • Other (please specify)

1. GIT: GIT scoring assessment, other (free text):
2. GIT: Please describe any data/notes not already listed that you think are important for interpretation of the data (if there is no additional information that you wish to explain, please leave this question blank) (free text):

Section 12: MODS

1. MODS: Number of patients included in MODS assessment (free text):
2. MODS: Score/assessment tool under study (AKA risk factor) (select all that apply):

• NPMOD, P-MODS • PELOD • PODIUM • Organ Failure Index (OFI) • PRISM • PRISM II • PRISM III • PRISM IV • O-PRISM • PIM • PIM II • PIM III • International Pediatric Sepsis Consensus (IPCC)/Goldstein • Phoenix Criteria • pSOFA • Graft vs. Host Disease • Cytokine release syndrome • Early warning system • Other definition of Sepsis/septic shock • Other (please specify)

1. MODS: MODS scoring assessment, other (free text):
2. MODS: Please describe any data/notes not already listed that you think are important for interpretation of the data (if there is no additional information that you wish to explain, please leave this question blank) (free text):

Note questions 89-120 repeat for all risk factors:

1. {organ system}, {risk factor}: timing of score/tool assessment/risk factor (e.g., day #1 of PICU admission, etc) (multiple choice):
    • At diagnosis • At hospital admission • At ICU admission • During ICU stay • Other (specify)
2. {organ system}, {risk factor}: timing other (free text):
3. {organ system}, {risk factor}: Outcomes assessed (check all that apply):

• Mortality • Duration of support or ICU care • Functional outcomes/residual morbidity • Organ-specific outcomes/residual morbidity • Outcomes related to MODS • Cost of medical care • Other patient-centered outcomes (e.g., symptom improvement, quality of life, quality of dying, effect on loved ones) • Other

1. {organ system}, {risk factor}, Mortality: Mortality (check all that apply) [shown if A is selected in Q50]:

• PICU mortality • 28-day mortality • Hospital (admission) mortality • Event-free survival • Overall survival • 100-day survival (typically post HCT) • 1-month mortality • 3-month survival/mortality (specify) • 6-month survival/mortality (specify) • 12-month survival/mortality (specify) • Other (specify)

1. {organ system}, {risk factor}, Mortality: Mortality, other (free text) [shown if A is selected in Q50]:
2. {organ system}, {risk factor}, Mortality: What statistical test(s) were used in the analysis between this risk factor and outcome(s) (free text: from methods section) [shown if A is selected in Q50]:
3. {organ system}, {risk factor}, Mortality: (1) Please describe the identified statistically significant and insignificant relationship(s) between this risk factor and outcome(s), (2) the location this information is found (e.g. Table 3, Figure 1, etc), and (3) the cutoff for continuous values if determined (free text) [shown if A is selected in Q50]:
4. {organ system}, {risk factor}, Duration of support: Duration of support or ICU care (check all that apply) [shown if B is selected in Q50]:

• Duration of mechanical support • Duration of ICU stay • Duration of renal replacement therapy • Duration of vasopressor use • Duration of hospital stay • Hospital-free days • ICU-free days • Vasopressor-free days • Mechanical ventilation-free days • Renal replacement-free days • Days alive without life support • Other (specify)

1. {organ system}, {risk factor}, Duration of support: Duration of support, other (free text) [shown if B is selected in Q50]:
2. {organ system}, {risk factor}, Duration of support: What statistical test(s) were used in the analysis between this risk factor and outcome(s) (free text: from methods section) [shown if B is selected in Q50]:
3. {organ system}, {risk factor}, Duration of support: (1) Please describe the identified statistically significant and insignificant relationship(s) between this risk factor and outcome(s), (2) the location this information is found (e.g. Table 3, Figure 1, etc), and (3) the cutoff for continuous values if determined (free text) [shown if B is selected in Q50]:
4. {organ system}, {risk factor}, Functional Outcomes: Functional outcomes /residual morbidity (check all that apply) [shown if C is selected in Q50]:

• Functional Outcomes Measures (Quantitative Functional Status Scale (FSS), Pediatric Cerebral Performance Category (PCPC); Pediatric Overall Performance Category (POPC); Pediatric Evaluation of Disability Inventory (PEDI); Functional Independence Measure for Children (WeeFIM), etc.) • Cognitive Outcome Measures (e.g., Mullen Scales of Early Learning (MSEL); Weschler Intelligence Scale for Children (WISC); Bayley Scales of Infant and Toddler Development (BSID), etc.) • Adaptive Outcome Measures (e.g. Vineland Adaptive Behavior Scales (VASB), etc.) • Development of associated mental illness (e.g. depression, post-traumatic stress disorder, acute stress disorder, anxiety disorder) • Other (please specific)

1. {organ system}, {risk factor}, Functional Outcomes: Functional Outcomes, other (free text) [shown if C is selected in Q50]:
2. {organ system}, {risk factor}, Functional Outcomes: What statistical test(s) were used in the analysis between this risk factor and outcome(s) (free text: from methods section) [shown if C is selected in Q50]:
3. {organ system}, {risk factor}, Functional Outcomes: (1) Please describe the identified statistically significant and insignificant relationship(s) between this risk factor and outcome(s), (2) the location this information is found (e.g. Table 3, Figure 1, etc), and (3) the cutoff for continuous values if determined (free text) [shown if C is selected in Q50]:
4. {organ system}, {risk factor}, Organ Specific Outcomes: Organ Specific Outcomes (check all that apply) [shown if D is selected in Q50]:

• Chronic mechanical ventilation (including CPAP, BiPAP, mechanical ventilation; i.e. required outside of ICU admission or hospital admission) • Need for tracheostomy • Need for gastric tube • Chronic oxygen requirement (i.e. required outside hospital admission) • Other (please specify)

1. {organ system}, {risk factor}, Organ Specific Outcomes: Organ Specific Outcomes, other (free text) [shown if D is selected in Q50]:
2. {organ system}, {risk factor}, Organ Specific Outcomes: What statistical test(s) were used in the analysis between this risk factor and outcome(s) (free text: from methods section) [shown if D is selected in Q50]:
3. {organ system}, {risk factor}, Organ Specific Outcomes: (1) Please describe the identified statistically significant and insignificant relationship(s) between this risk factor and outcome(s), (2) the location this information is found (e.g. Table 3, Figure 1, etc), and (3) the cutoff for continuous values if determined (free text) [shown if D is selected in Q50]:
4. {organ system}, {risk factor}, Multi-Organ Dysfunction: Outcomes related to MODS (check all that apply) [shown if E is selected in Q50]:

• Duration of new or progressive MODS (NPMODS) • Composite time to complete organ dysfunction resolution • 28-day organ dysfunction score (i.e., PELOD-2, PRISM, etc.) • Other (please specify)

1. {organ system}, {risk factor}, Multi-Organ Dysfunction: Outcomes related to MODS, other (free text) [shown if E is selected in Q50]:
2. {organ system}, {risk factor}, Multi-Organ Dysfunction: What statistical test(s) were used in the analysis between this risk factor and outcome(s) (free text: from methods section) [shown if E is selected in Q50]:
3. {organ system}, {risk factor}, Multi-Organ Dysfunction: (1) Please describe the identified statistically significant and insignificant relationship(s) between this risk factor and outcome(s), (2) the location this information is found (e.g. Table 3, Figure 1, etc), and (3) the cutoff for continuous values if determined (free text) [shown if E is selected in Q50]:
4. {organ system}, {risk factor}, Cost of medical care: Cost of medical care (free text) [shown if F is selected in Q50]:
5. {organ system}, {risk factor}, Cost of medical care: What statistical test(s) were used in the analysis between this risk factor and outcome(s) (free text: from methods section) [shown if F is selected in Q50]:
6. {organ system}, {risk factor}, Cost of medical care: (1) Please describe the identified statistically significant and insignificant relationship(s) between this risk factor and outcome(s), (2) the location this information is found (e.g. Table 3, Figure 1, etc), and (3) the cutoff for continuous values if determined (free text) [shown if F is selected in Q50]:
7. {organ system}, {risk factor}, Other patient center outcomes: Other patient center outcomes (free text) [shown if G is selected in Q50]:
8. {organ system}, {risk factor}, Other patient center outcomes: What statistical test(s) were used in the analysis between this risk factor and outcome(s) (free text: from methods section) [shown if G is selected in Q50]:
9. {organ system}, {risk factor}, Other patient center outcomes: (1) Please describe the identified statistically significant and insignificant relationship(s) between this risk factor and outcome(s), (2) the location this information is found (e.g. Table 3, Figure 1, etc), and (3) the cutoff for continuous values if determined (free text) [shown if G is selected in Q50]:
10. {organ system}, {risk factor}, Other center outcomes: Other patient center outcomes (free text) [shown if H is selected in Q50]:
11. {organ system}, {risk factor}, Other center outcomes: What statistical test(s) were used in the analysis between this risk factor and outcome(s) (free text: from methods section) [shown if H is selected in Q50]:
12. {organ system}, {risk factor}, Other center outcomes: (1) Please describe the identified statistically significant and insignificant relationship(s) between this risk factor and outcome(s), (2) the location this information is found (e.g. Table 3, Figure 1, etc), and (3) the cutoff for continuous values if determined (free text) [shown if H is selected in Q50]:

Section 13: Bias Assessment (QUIPS):

Domain 1, Study Participation

1. Source of Target Population: The source population or population of interest is adequately described for key characteristics (e.g., for children with cancer, inpatient, and within a year of cancer treatment completion) (multiple choice):

• Yes • Partial • No • Unsure

1. Method used to Identify Population: The sampling frame and recruitment are adequately described, including methods to identify the sample sufficient to limit potential bias (number and type used, e.g., referral patterns in health care) (multiple choice):

• Yes • Partial • No • Unsure

1. Recruitment Period: Period of recruitment is adequately described (multiple choice):

• Yes • Partial • No • Unsure

1. Place of Recruitment: Place of recruitment (setting and geographic location) are adequately described (multiple choice):

• Yes • Partial • No • Unsure

1. Inclusion and Exclusion Criteria: "Inclusion and exclusion criteria are adequately described (e.g., including explicit diagnostic criteria or "zero time" description)." (multiple choice):

• Yes • Partial • No • Unsure

1. Adequate Study Participation: There is adequate participation in the study by eligible individuals (multiple choice):

• Yes • Partial • No • Unsure

1. Baseline Characteristics: The baseline study sample (e.g., individuals entering the study) is adequately described including age, sex, oncologic diagnosis, and if patient post HSCT, underlying indication (malignant vs. nonmalignant) for HSCT (multiple choice):

• Yes • Partial • No • Unsure

1. Source of Target Population: The source population or population of interest is adequately described for key characteristics (e.g., for children with cancer, inpatient, and within a year of cancer treatment completion) (multiple choice):

• Yes • Partial • No • Unsure

1. Source of Target Population: The source population or population of interest is adequately described for key characteristics (e.g., for children with cancer, inpatient, and within a year of cancer treatment completion) (multiple choice):

• Yes • Partial • No • Unsure

1. Source of Target Population: The source population or population of interest is adequately described for key characteristics (e.g., for children with cancer, inpatient, and within a year of cancer treatment completion) (multiple choice):

• Yes • Partial • No • Unsure

1. Source of Target Population: The source population or population of interest is adequately described for key characteristics (e.g., for children with cancer, inpatient, and within a year of cancer treatment completion) (multiple choice):

• Yes • Partial • No • Unsure

1. Source of Target Population: The source population or population of interest is adequately described for key characteristics (e.g., for children with cancer, inpatient, and within a year of cancer treatment completion) (multiple choice):

• Yes • Partial • No • Unsure

Domain 2: Study Attrition

1. Proportion of Baseline Sample Available for Analysis: Response rate (e.g., proportion of study sample completing the study and providing outcome data) is adequate. (multiple choice):

• Yes • Partial • No • Unsure

1. Attempts to Collect Information on Participants Who Dropped Out: Attempts to collect information on participants who dropped out of the study are described. (multiple choice):

• Yes • Partial • No • Unsure

1. Reasons and Potential Impact of Subjects Lost to Follow-Up: Reasons for loss to follow-up are provided. (multiple choice):

• Yes • Partial • No • Unsure

1. Outcome and risk factor information on those lost to follow-up: Participants lost to follow-up are adequately described for key characteristics (e.g., oncologic disease/severity characteristics, organ failure severity/characteristics, possible perceived futility of treatment, and demographics, including ability to pay for treatment). (multiple choice):
    • Yes • Partial • No • Unsure
2. Outcome and risk factor information on those lost to follow-up: There are no important differences between key characteristics (e.g., oncologic disease/severity characteristics, organ failure severity/characteristics, possible perceived futility of treatment, and demographics, including ability to pay for treatment) and outcomes in participants who completed the study and those who did not. (multiple choice):

• Yes • Partial • No • Unsure

Domain 3: Risk Factor Management

1. Definition of the Risk Factor: A clear definition or description of 'risk factor' is provided (e.g., such as adequate definition or defined threshold for the risk factor or organ failure/dysfunction). (multiple choice):

• Yes • Partial • No • Unsure

1. Valid and Reliable Measurement of Risk Factor: Method of risk factor measurement is adequately valid and reliable to limit misclassification bias (e.g., may include relevant outside sources of information on measurement properties, also characteristics, such as blind measurement and limited reliance on recall). (multiple choice):

• Yes • Partial • No • Unsure

1. Valid and Reliable Measurement of Risk Factor: Continuous variables are reported or appropriate cut-points (e.g., not data-dependent) are used. (multiple choice):

• Yes • Partial • No • Unsure

1. Method and Setting of Risk Factor Measurement: The method and setting of measurement of risk factor is the same for all study participants. (multiple choice):

• Yes • Partial • No • Unsure

1. Proportion of data on Risk Factor Available for Analysis: Adequate proportion (>80%) of the study sample has complete data for risk factor variable. (multiple choice):

• Yes • Partial • No • Unsure

1. Method Used for Missing Data: Appropriate methods of imputation are used for missing risk factor data. (multiple choice):

• Yes • Partial • No • Unsure

Domain 4, Outcome Measurement

1. Definition of the Outcome: A clear definition of outcome is provided, including duration of follow-up and level and extent of the outcome construct. (multiple choice):

• Yes • Partial • No • Unsure

1. Valid and Reliable Measurement of Outcome: The method of outcome measurement used is adequately valid and reliable to limit misclassification bias (e.g., may include relevant outside sources of information on measurement properties, also characteristics, such as blind measurement and confirmation of outcome with valid and reliable test). (multiple choice):

• Yes • Partial • No • Unsure

1. Method and Setting of Outcome Measurement: The method and setting of outcome measurement is the same for all study participants. (multiple choice):

• Yes • Partial • No • Unsure

Domain 5, Study Confounding

1. Important Confounders Measured: All important confounders are measured when appropriate (e.g., age, sex, cancer treatment (e.g. chemotherapeutic regimen, HSCT conditioning), oncologic diagnosis, disease severity (grading, risk stratification, tumor burden), Disease status (e.g. active disease, relapse/refractor disease, etc.) HSCT type). (multiple choice):

• Yes • Partial • No • Unsure

1. Definition of the Confounding Factor: Clear definitions of the important confounders measured are provided (e.g., chemotherapeutic regimens, oncologic disease severity [see above], baseline values or other measures of organ dysfunction/failure). (multiple choice):

• Yes • Partial • No • Unsure

1. Valid and Reliable Measurement of Confounders: Measurement of all important confounders is adequately valid and reliable (e.g., may include relevant outside sources of information on measurement properties, also characteristics, such as blind measurement and limited reliance on recall). (multiple choice):

• Yes • Partial • No • Unsure

1. Method and Setting of Confounding Measurement: The method and setting of confounding measurement are the same for all study participants. (multiple choice):

• Yes • Partial • No • Unsure

1. Method used for Missing Data: Appropriate methods are used if imputation is used for missing confounder data. (multiple choice):

• Yes • Partial • No • Unsure

1. Appropriate Accounting for Confounding: Important potential confounders are accounted for in the study design (e.g., matching for key variables, stratification, or initial assembly of comparable groups). (multiple choice):
    • Yes • Partial • No • Unsure
2. Appropriate Accounting for Confounding: Important potential confounders are accounted for in the analysis (i.e., appropriate adjustment). (multiple choice):

• Yes • Partial • No • Unsure

Domain 6, Statistical Analysis and Presentation

1. Presentation of Analytical Strategy: There is sufficient presentation of data to assess the adequacy of the analysis. (multiple choice):

• Yes • Partial • No • Unsure

1. Model Development Strategy: The strategy for model building (e.g., inclusion of variables in the statistical model) is appropriate and is based on a conceptual framework or model. (multiple choice):

• Yes • Partial • No • Unsure

1. Model Development Strategy: The selected statistical model is adequate for the design of the study. (multiple choice):

• Yes • Partial • No • Unsure

1. Reporting of Results: There is no selective reporting of results. (multiple choice):

• Yes • Partial • No • Unsure
